# Bactericidal Effect of Lauric Acid-Loaded PCL-PEG-PCL Nano-Sized Micelles on Skin Commensal *Propionibacterium acnes*

**DOI:** 10.3390/polym8090321

**Published:** 2016-08-27

**Authors:** Thi-Quynh-Mai Tran, Ming-Fa Hsieh, Keng-Lun Chang, Quoc-Hue Pho, Van-Cuong Nguyen, Ching-Yi Cheng, Chun-Ming Huang

**Affiliations:** 1 International Master Program of Biomedical Material and Technology, Chung Yuan Christian University, 200 Chung-Pei Road, Chung Li District, Taoyuan City 32023, Taiwan; ttqm1992@gmail.com; 2 Department of Biomedical Engineering, Chung Yuan Christian University, 200 Chung-Pei Road, Chung Li District, Taoyuan City 32023, Taiwan; noask518@gmail.com (K.-L.C.); phoquochue@gmail.com (Q.-H.P.); 3 Center for Biomedical Technology, Chung Yuan Christian University, 200 Chung-Pei Road, Chung Li District, Taoyuan City 32023, Taiwan; 4 Department of Chemical Engineering, Industrial University of Ho Chi Minh City, 12 Nguyen Van Bao St., Go Vap, Ho Chi Minh City 7000, Vietnam; nvc@iuh.edu.vn; 5 Graduate Institute of Health Industry Technology and Research Center for Industry of Human Ecology, Chang Gung University of Science and Technology, No. 261, Wenhua 1st Road, Guishan District, Taoyuan City 33303, Taiwan; Jennycheng@mail.cgust.edu.tw; 6 Department of Dermatology, School of Medicine, University of California, San Diego, CA 92121, USA; chunming@ucsd.edu

**Keywords:** poly(ε-caprolactone)-poly(ethylene glycol)-poly(ε-caprolactone), lauric acid, micelles, *Propionibacterium acnes*, drug delivery system, nanoparticles

## Abstract

Acne is the over growth of the commensal bacteria *Propionibacterium acnes* (*P. acnes*) on human skin. Lauric acid (LA) has been investigated as an effective candidate to suppress the activity of *P. acnes*. Although LA is nearly insoluble in water, dimethyl sulfoxide (DMSO) has been reported to effectively solubilize LA. However, the toxicity of DMSO can limit the use of LA on the skin. In this study, LA-loaded poly(ɛ-caprolactone)-poly(ethylene glycol)-poly(ɛ-caprolactone) micelles (PCL-PEG-PCL) were developed to improve the bactericidal effect of free LA on *P. acnes*. The block copolymers mPEG-PCL and PCL-PEG-PCL with different molecular weights were synthesized and characterized using ^1^H Nuclear Magnetic Resonance spectroscopy (^1^H NMR), Fourier-transform infrared spectroscopy (FT-IR), Gel Permeation Chromatography (GPC), and Differential Scanning Calorimetry (DSC). In the presence of LA, mPEG-PCL diblock copolymers did not self-assemble into nano-sized micelles. On the contrary, the average particle sizes of the PCL-PEG-PCL micelles ranged from 50–198 nm for blank micelles and 27–89 nm for LA-loaded micelles. The drug loading content increased as the molecular weight of PCL-PEG-PCL polymer increased. Additionally, the minimum inhibitory concentration (MIC) and the minimum bactericidal concentration (MBC) of free LA were 20 and 80 μg/mL, respectively. The MICs and MBCs of the micelles decreased to 10 and 40 μg/mL, respectively. This study demonstrated that the LA-loaded micelles are a potential treatment for acne.

## 1. Introduction

Acne is the most common skin disease that appears usually in adolescence and adulthood. It is caused by the gram-positive bacterium *Propionibacterium acnes* (*P. acnes*) and mostly affects skin with many sebaceous glands or hair follicles. *P. acnes* induces monocytes to secrete pro-inflammatory cytokines, such as interleukin-1β, interleukin-8, and interleukin-12, which then contribute to the inflammation [1,2,3,4]. The conventional antibiotic retinoid can ease acne symptoms for most patients, but contraindications in pregnant or lactating women limit their use [3,5]. Various antimicrobial agents and antibiotics have been developed to treat acne. Among those drugs, benzoyl peroxide (BPO) has been reported as one of the most important agents for reducing the acne vulgaris. However, BPO produces high erythema, scaling, and burning [3]. For that reason, attempts have been made to find new anti-acne agents with both excellent therapeutic efficacy and negligible adverse side effects [5].

Short-chain fatty acids (SCFAs) have been known to exhibit self-antimicrobial activity to prevent microbial colonization. Previous studies of various SCFAs have demonstrated their antibacterial efficacy [2,6]. For example, oleic acid was reported to inhibit the growth of *Staphylococcus aureus* in a mouse model, and linoleic acid overcame antibiotic resistance developed by various clinically isolated strains of *Helicobacter pylori* [6]. In 2009, lauric acid (LA) was reported as a bactericidal agent for acne less harmful than benzoyl peroxide (BPO) [2,3,7]. However, the use of LA is limited due to its low solubility in water. Although dimethyl sulfoxide (DMSO) has been reported to improve the solubility of LA, the cytotoxicity of DMSO can limit its usefulness in the treatment of acne [3].

Recent studies of drug-loaded polymeric micelles showed enhanced characteristics of these materials, such as antibacterial efficiency, stability, drug solubility, and selective targeting [8,9]. Poly(ε-caprolactone) (PCL) has been studied as an ideal material for drug delivery because of its adjustable degradation time [8,10,11]. On the contrary, poly(ethylene glycol) (PEG) is a water-soluble, non-toxic molecule that has no antigenicity immunogenicity and can thus co-polymerize with ε-caprolactone (ε-CL) to take advantage of its improved bioavailability and biodegradation as well as mild toxicity [8,10,11,12].

Amphiphilic block copolymers have received particular interest as drug delivery systems because of their ability to self-assemble into nano-sized micelles. Various structures of block copolymers have been used to encapsulate hydrophobic drugs, such as AB diblock copolymers where the A block of the hydrophobic segment is at the core, and the B block of the hydrophilic segment is distributed on the outer surface of the micelles [13]. However, most drugs encapsulated in this type of polymeric micelle are anticancer drugs such as doxorubicin. The challenges to incorporate fatty acids or lipids arise from the incompatibility of the hydrocarbon chain to the PCL segment in the core of the micelles. Recently, Hallouard et al. reported iodinated oil-loaded PCL/PCL-mPEG formulation for the application of computerized tomography contrast agent [14]. To prepare stable formulations, the PCL-mPEG diblock copolymer was mixed with PCL in their study. As such, the hydrophobic phase in the formulation plays a pivotal role in stabilizing lipid drug encapsulation in the nanoparticles. ABA triblock copolymers, such as PCL-PEG-PCL, are variants of the diblock copolymers where the composition of the hydrophobic phase is increased in the formulation compared with that of a drug carrier using the AB diblock copolymer.

In this study, we synthesized PCL-PEG-PCL copolymers as drug carriers of LA. For comparison, mPEG-PCL diblock copolymers were also studied. In aqueous medium, this copolymer can form nano-sized micelles. The physical properties of the blank and LA-loaded micelles were characterized, including particle size and zeta potential. The minimum inhibitory concentration (MIC) and the minimum bactericidal concentration (MBC) of free LA and LA-loaded micelles on *P. acnes* were determined.

## 2. Experimental Section

### 2.1. Materials

Poly(ethylene glycol) (PEG, *M*_n_ = 4000) was purchased from Showa (Tokyo, Japan); ε-caprolactone (ε-CL), polymerization catalyst Sn(Oct)_2_, dimethyl sulfoxide (DMSO), 1,6-diphenyl-1,3,5-hexatriene (DPH), 3-(4,5-dimethylthiazol-2-yl)-2,5-diphenyltetrazolium bromide (MTT), Noble agar, phosphotungstic acid, potassium bromide (KBr), potassium phosphate monobasic, sodium chloride, and sodium phosphate were all purchased from Sigma-Aldrich Chem. Inc. (St. Louis, MO, USA). Acetone, acetonitrile (ACN), d-chloroform (CDCl_3_), dichloromethane, ethyl ether, ethanol, hexane, and tetrahydrofuran were provided by ECHO Chemicals (Miaoli, Taiwan).

### 2.2. Synthesis of PCL-PEG-PCL Copolymers

The PCL-PEG-PCL copolymer (named as PCEC) was synthesized by a ring-opening polymerization reaction as previously described with some modifications [11]. The molecular weights of the PCEC copolymers were designed for 8000, 14,000, and 24,000 Da, corresponding to PC_20_E_40_C_20_, PC_50_E_40_C_50_, and PC_100_E_40_C_100_, respectively. The ratios of the molecular weights of the hydrophilic block to the hydrophobic block were 1:1, 1:2.5, and 1:5, respectively. Specifically, to synthesize PC_20_E_40_C_20_, a three-necked round bottom flask was purged by a continuous flow of nitrogen gas and heated at 90 °C. Then, 10 g of PEG (*M*_w_ = 4000) and 0.146 mL of catalyst Sn(Oct)_2_(1 molar% of ɛ-CL) were introduced into the flask under mechanical stirring for 30 min. After the temperature was increased to 130 °C, 10 g of ε-CL was added to the flask for a 12-h reaction [11,15]. The resulting product was dissolved in dichloromethane and precipitated in diethyl ether:*n*-hexane at a volumetric ratio of 7:3. This step was repeated several times to purify the product. Finally, the product was filtered and dried under a vacuum for 24 h.

### 2.3. Characterization of the PCL-PEG-PCL Copolymers

After synthesis of the PCEC triblock copolymers, the resulting samples were characterized by different physicochemical methods. The PCEC vibrational spectra were measured using a Fourier-transform infrared (FT-IR) spectrometer (model 410, JASCO, Tokyo, Japan) in the range of 4000 to 400 cm^−1^. The samples were compressed with KBr for spectral measurements. The copolymer chemical structure was determined by ^1^H Nuclear Magnetic Resonance spectroscopy (^1^H NMR) using CDCl_3_ as a solvent operating at 500 MHz (Bruker, Billerica, MA, USA). The molecular weight of the copolymers was estimated by calculating the integrated peak area of the mPEG and PCL segments from the ^1^H NMR spectra. In addition, Gel Permeation Chromatography (GPCmax, Viscotek, Houston, TX, USA) was used to measure the molecular weights and the distribution of the PCEC triblock copolymers using THF as the mobile phase. Polystyrene (PS) standards were used to calibrate the number-average and weight-average molecular weights. The thermal properties (melting/crystallization) of the copolymers were investigated by Differential Scanning Calorimetry (Jade DSC, PerkinElmer, Waltham, MA, USA) with a range of heating temperatures from 20 to 100 °C under a nitrogen atmosphere at a heating and cooling rate of 5 °C/min.

### 2.4. Preparation of Blank or LA-Loaded PCL-PEG-PCL Nano-Sized Micelles

Micelle preparation was carried out by following the thin film hydration method as reported elsewhere [16]. Briefly, various amounts of PCEC and LA were co-dissolved in ACN at different ratios as shown in Table 1. The resulting solutions were rigorously mixed by a vortex. Afterwards, ACN was removed by a rotary evaporator at 60 °C, and 2 mL of deionized water kept at 80 °C was then added rapidly to the dried samples in an ultrasonic bath for 10 min. After stirring for 3 h at room temperature, the micellar solution was centrifuged (2500 g, 10 min) and filtered through a 0.45-μm filter. Particle size and zeta potential of the micelles were measured using a zeta potential analyzer (90Plus, Brookhaven Instruments Corporation, Holtsville, NY, USA). The morphology of the prepared micelles was observed on a Transmission electron microscope (TEM) (JEM2000FXII, JOEL, Tokyo, Japan), and 0.5 mg/mL micellar solutions were spotted on a copper grid. The samples were negatively stained with phosphotungstic acid (0.1 wt %) and dried at room temperature.

### 2.5. Critical Micelle Concentration (CMC)

The CMC of the copolymer was measured by the pyrene 1:3 method using DPH as a fluorescent probe [3,17]. The polymeric micelles were prepared to obtain a range of concentrations between 1.2 × 10^−3^ and 2 mg/mL. Aliquots (2 mL) of the polymeric micelles were added to 20 µL of DPH (0.4 mM in MeOH) to give a 4 × 10^−6^ M DPH/copolymer solution. The resultant solution was incubated in the dark for 5 h. The intensities *I*_1_ and *I*_3_ were measured at wavelengths corresponding to the first and third vibronic bands at 373 and 384 nm [17]. The CMC of the copolymer was determined based on plots of *I*_1_/*I*_3_ ratio and concentrations.

### 2.6. Loading Content of LA in the Micelles

To determine the loading content of LA in the micelles, the samples were first dried by rotary evaporation following by dissolving in methanol and chemically deriving with naphthacyl ester [3,18]. Briefly, 100 mg of KHCO_3_ was added into 1 mL of as-prepared micelles and dried overnight. Subsequently, the samples were mixed with a solution of 20 mM 2-bromo-2′-acetonaphthone and 4.54 mM 18-crown-6 and then kept at 80 °C for 30 min using a Thermomixer (Eppendorf 5810, Westbury, NY, USA). The resulting solutions were measured by reversed-phase high performance liquid chromatography (HPLC) (Alliance 2695 separation module, Waters, Milford, MA, USA) using a Nova-Pak C18 column (4 µm, 4.6 mm× 250 mm, Waters) and a guard column (Nova-Pak C18; 4 µm, 3.9 mm× 20 mm, Waters). The column temperature was maintained at 30 °C with a solvent gradient of methanol/acetonitrile/water ranging from 80:10:10 (*v*/*v*/*v*) that was increased linearly to 90:10:0 (*v*/*v*/*v*). Derived LA was detected by UV/VIS absorbance at a wavelength of 254 nm at 20 °C. The concentration of LA was calculated according to the following equations that had been produced from the calibration curve of free LA.

(1) $$\text{Drug loading content }(\text{DLC})=\frac{\text{Amount of LA in micelles}}{\text{Amount of polymer}+\text{LA}}\times 100\%$$

### 2.7. Preparation of Bacteria for Antibacterial Testing

In this study, *Propionibacterium acnes* (*P. acnes*) (BCRC 10723) was purchased from the Bioresource Collection and Research Center (Hsinchu, Taiwan). *P. acnes* was cultured in Reinforced Clostridial Medium (RCM) (OXOID, Hampshire, UK) under anaerobic conditions using Gas-Pak (AnaeroPack-Anaero, Mitsubishi Gas Chemical Co., Tokyo, Japan) with rotary shaking at 37 °C. Consequently, the culture was maintained until it reached an OD_600_ value of 0.7. The bacteria were harvested by centrifugation (5000 g for 10 min) and then washed with sterile PBS and suspended in an appropriate amount of PBS for further experiments.

### 2.8. Minimum Inhibitory Concentration of the Micelles

MIC is the lowest concentration of an antimicrobial agent necessary to prevent bacterial growth. In our study, the concentration of free LA and micelles that inhibited 50% of *P. acnes* (MIC_50_) was determined using the two-fold serial dilution method [19,20]. The cultured bacteria were measured by spectrophotometer at an absorbance wavelength of 600 nm and a concentration of 2 × 10^6^ (CFU/mL). In a 96-well plate, 100 μL of the original bacterial suspension was pipetted into the first well. Two-fold serial dilutions were made from the 2nd to 11th wells with 50 µL of PBS, and thus the concentration of bacteria was 10^6^ CFU/mL, which was then incubated with LA or LA-loaded micelles (0–100 µg/mL) in PBS under aerobic conditions for 24 h. The control received only 5% DMSO. Later on, the absorbance at 600 nm was measured to estimate bacterial growth.

### 2.9. The Minimum Bactericidal Concentration of Micelles

MBC is the lowest concentration of an antibacterial agent that reduces the viability of the initial bacterial inoculum by ≥99.9%. It can be determined by the following two steps: incubation in RCM broth (19 g RCM in 500 mL double-distilled water) and then sub-culture on RCM agar plates (19 g of RCM and 7.5 g of agar in 500 mL of DD water). To determine the MBC of free LA or LA-loaded micelles against *P. acnes*, 1 × 10^7^ CFU/mL *P. acnes* was incubated with various concentrations of LA or LA-loaded micelles (0–100 µg/mL) in PBS at 37 °C for 5 h under anaerobic conditions, and 5% DMSO in PBS was used as a control. The mixture then was diluted from 10^−1^ to 10^−6^ times in PBS. Five microliters of the diluted solutions were spotted on RCM agar plates, which were then incubated at 37 °C for 96 h. The number of colony forming units (CFUs) was calculated using the following equation [19,21]:

(2) $$\text{CFU}=\frac{\text{Number of colonies counted}}{\text{Amount plates }(\text{in mL})\times \text{dilution}}$$

## 3. Results and Discussion

### 3.1. Characterization of PCL-PEG-PCL Copolymers (PCECs)

**Figure 1:**
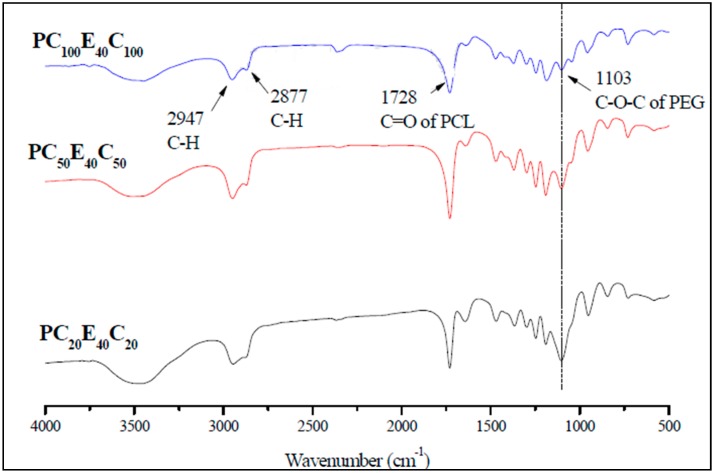
Fourier-transform infrared spectra of three PCL-PEG-PCL copolymers. The polymers were pelleted with KBr for transmission measurement. The *Y*-axis indicates the transmittance (%).

PCECs were synthesized with three different molecular weights. The PEG, which functioned as the central segment, had a molecular weight of 4000 Da, and the theoretical molecular weight of PCL segments at both ends, as displayed in Table 2, were 4000, 10,000, and 20,000 Da. The FT-IR spectra of the PCECs are shown in Figure 1. The results demonstrated that the peak of C–H stretching appeared at 2947 and 2877 cm^−1^, which proved the existence of PCL segments in the copolymer. A C=O stretching band was observed at 1728 cm^−1^, which can be attributed to the PCL segment, and another peak appeared at 1103 cm^−1^ for C–O–C stretching vibration of the repeated –OCH_2_CH_2_ units of PEG [22,23,24,25]. In addition, those peaks were also found in the spectra of PC_20_E_40_C_20_ and PC_50_E_40_C_50_. In Figure 1, when the molecular weight of the PCL segment was increased, the intensity of the peak at 1103 cm^−1^ was slightly decreased with respect to 1728 cm^−1^, which was the C=O of PCL. That is because the more abundant amount of PCL in PCECs led to pronounced areas of C=O stretching peak.

According to the ^1^HNMR spectra of PCECs, the chemical structure of PCECs was determined (Figure S1). Protons of the PEG segment were investigated at δ = 3.6 ppm, whereas the chemical shifts of protons in the PCL segment were δ = 2.3, 1.6, 1.3, and 4.0 ppm [26]. To estimate the molecular weights of PCECs, the areas under the peak in ^1^H NMR spectra were used. Based on the known molecular weights of PEG (4000 Da), the peak areas of each hydrogen on the polymer were obtained. After that, the molecular weights of the polymerized PCL segments were calculated. In addition to the molecular weights estimated by ^1^H NMR, the molecular weights measured by GPC are also summarized in Table 2. According to the molecular weights estimated by ^1^HNMR, the molecular weights of PC_20_E_40_C_20_, PC_50_E_40_C_50,_ and PC_100_E_40_C_100_ were close to the theoretical values. In GPC analysis, the number of average molecular weight (*M*_n_) and average molecular weight (*M*_w_) deviated from the theoretical values because the conventional standard (polystyrene) was used. However, the polydispersity indexes of the PCECs were 1.17, 1.29, and 1.33. This indicates that the molecular weights of PCECs were narrowly distributed, especially for PC_20_E_40_C_20_.

Differential Scanning Calorimetry (DSC) was used to characterize the thermal properties of the synthesized copolymers. The thermograms for the second heating/cooling curves were recorded and are displayed in Figure S2. For PC_20_E_40_C_20_ and PC_50_E_40_C_50_, the thermograms showed double peaks, indicating two melting temperatures (*T*_m_) assigned for PEG and PCL, respectively. The higher melting temperatures of PC_20_E_40_C_20_ and PC_50_E_40_C_50_ were determined to be 49.41 and 51.39 °C, respectively, which corresponded to the melting point of the PCL segment in the crystal phase. Although the PCL-PEG-PCL was a copolymer, the segments PEG and PCL segments remained phase separated. Therefore, bimodal melting points were observed in PC_20_E_40_C_20_ and PC_50_E_40_C_50_ [27]. The *T*_m_ of PEG was relatively decreased along with the increase in the molecular weight of the PCECs. By contrast, there was only a single melting point at 54.22 °C for PC_100_E_40_C_100_, indicating the cooperative crystallization of PEG and PCL segments and/or the strong crystallization of the PCL segment. As a result, the PEG peak disappeared. The increased melting point is alternative evidence to demonstrate the increased molecular weight of the PCECs.

### 3.2. Characterization of PCL-PEG-PCL Micelles

#### 3.2.1. The Critical Micelle Concentration of PCL-PEG-PCL

**Figure 2:**
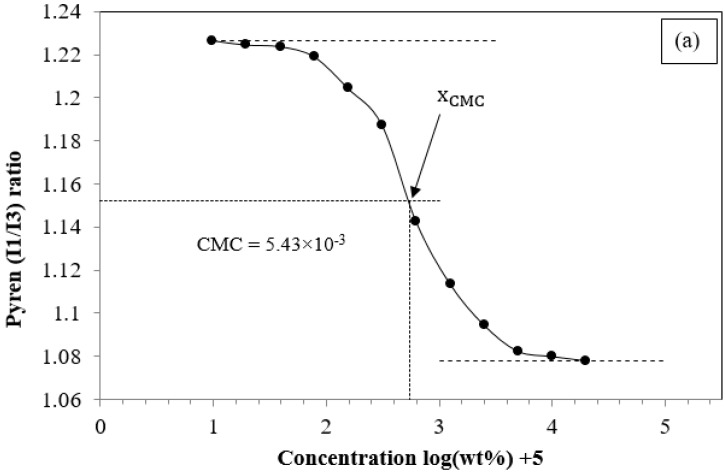

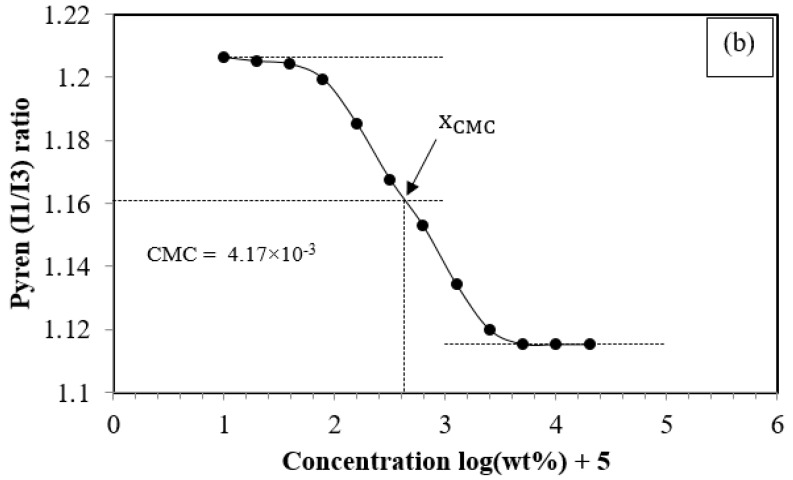

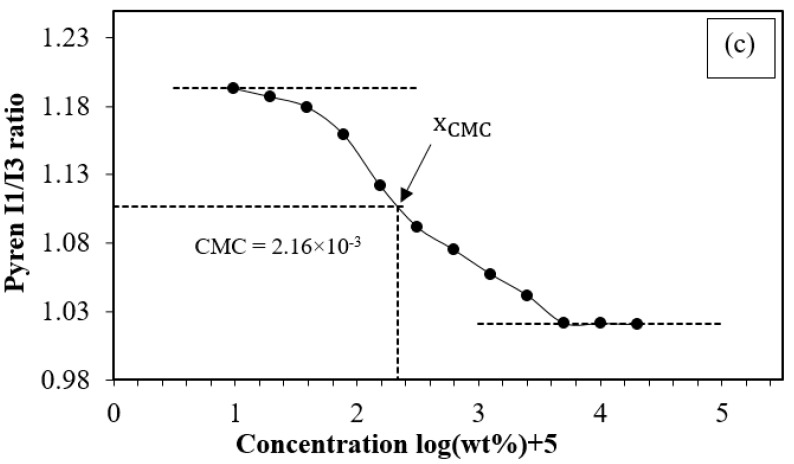
Measurement of critical micelle concentration (CMC) values for (**a**) PC_20_E_40_C_20_; (**b**) PC_50_E_40_C_50_; (**c**) PC_100_E_40_C_100_.

The CMC is used to estimate the lowest formation concentration of the micelles in water. The CMC is a particularly important parameter for micelle-based drug delivery systems to avoid burst release upon injection into the bloodstream. When the concentration of the amphiphilic block copolymers is below the CMC, it becomes thermodynamically unstable [28]. Determination of the CMCs for the PCECs was carried out by serial dilution of micellar solutions. Then, absorption of fluorescent probe (DPH) included in the cores of the micelles was recorded. We adopted the pyrene 1:3 ratio method to determine the CMC. The values of the micelles were determined based on the relationship of the intensity ratio of *I*_1_/*I*_3_ of pyrene included in the micelles and the concentrations. As shown in Figure 2, the CMC value was determined at the center point of the sigmoid. The CMC values of PC_20_E_40_C_20_, PC_50_E_40_C_50_, and PC_100_E_40_C_100_ (summarized in Table 2) were 5.43 × 10^−3^, 4.17 × 10^−3^, and 2.16 × 10^−3^ wt %, respectively. Apparently, the CMC values were reduced when the molecular weight (chain length) of the hydrophobic segment of PCL was increased from 2000 to 10000 Da [29]. This is because when the chain length of PCL is increased, the hydrophobic segments of PCL can pack more efficiently, and the hydrophobic interactions within the core of micelles are increased. Hence, the PCEC copolymers can more easily self-assemble in aqueous solution to form micelles, resulting in increased micelle stability in aqueous solutions at relatively lower concentrations [30]. Figure S3 displays the CMC values of diblock copolymers as 16.4 × 10^−3^, 8.91 × 10^−3^, and 4.47 × 10^−3^ wt % for PC_75_, PC_100_, and PC_150_, respectively. Figures S4–S6 display the physicochemical characterizations of diblock copolymers and Table S1 shows the summary of the results. Obviously, the increase in the hydrophobic segment (PCL in this study) leads to lower CMC values. Not only do the drug delivery systems for parental injection benefit from low CMC values but also those delivered through the skin can remain stable even after multiple doses [31]. Therefore, the results demonstrated that the stability of the micelle formed by triblock PCECs is more stable than that of diblock PCs.

**Figure 3:**
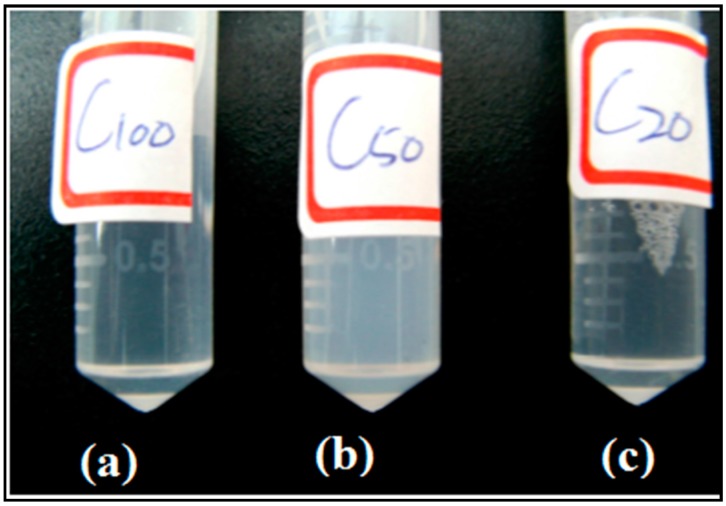
The results of hydration of the micelle aqueous solutions. (**a**) MC_100_; (**b**) MC_50_; (**c**) MC_20_.

As described in the experimental section, the micelle preparation method was similar to that of liposomes. During the hydration, the PCECs were added into hot water (80 °C), which was high enough to soften the polymers and allow them to easily self-assemble in aqueous solutions. As observed, the solution turned from transparent to turbid (shown in Figure 3) when hot water was added to the dried PCEC film.

#### 3.2.2. Particle Size and Surface Charges of the Micelles

The particle size and distribution of the MC micelles are displayed in Table 3. The average particle sizes were 50, 78, and 198 nm for MC_20_, MC_50,_ and MC_100_, respectively. When the ratio of PCEC to LA was 10:2, the particle sizes of MC_20_LA, MC_50_LA, and MC_100_LA were reduced to 27, 68, and 89 nm, respectively. For blank micelles, the greater the chain length of the PCL segment, the larger was the average particle size of the micelles. When LA was loaded onto the micelles, the average particle size of the micelles decreased. Previous studies on poly(lactic-*co*-glycolic acid) (PLGA) micelles loading caprylic acid (C8) [32] showed that caprylic acid could decrease the particle size of the micelles. Similar reports have indicated that the core of the micelle, in which the hydrophobic segment can accommodate a hydrophobic reagent, leads to a reduction in the particle size of the micelles [33].

Additionally, a similar result for particle size was also found in blank micelles of diblock copolymers (Figure S7c). The average diameters of the blank micelles for PC_75_, PC_100,_ and PC_150_ were 51, 61, and 88 nm, respectively. This demonstrated that particle sizes less than 100 nm can be easily fabricated using both the diblock and triblock copolymers synthesized in this study. However, we observed that LA-loaded micelles of diblock copolymers (PCs) have particle sizes over the nanoscale, e.g., all of them were micron-sized (Figure S7d). This dramatic change can also be observed under microscopy. Figure S7b indicates the morphology of PC particles along with oil droplets. Apparently, diblock copolymers of mPEG-PCL are difficult to self-assemble into stable micelles in the presence of LA, and, as a result, the suspensions were found (Figure S7a). As mentioned in the Introduction, the efficient loading of fatty acids or lipids requires higher loading of hydrophobic components of drug carriers, such as the addition of a single PCL into the formulation to form oil-loaded micelles [13,34,35].

**Figure 4:**
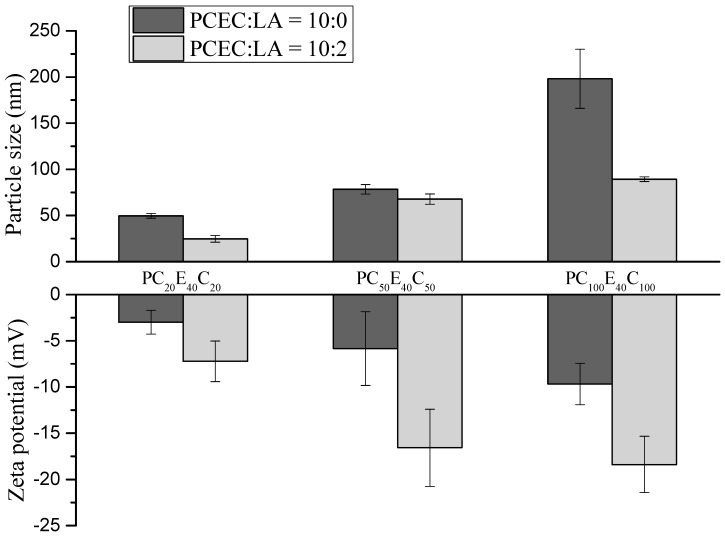
The average particle size and zeta potential of the micelles.

The surface charge of a micelle is one of the important factors in identifying the interactions of a micelle with bacteria, and it is typically measured as zeta-potential. Loading of a compound on to the micelle can cause the changes in the electrical potential profile on the micelle surface [3]. The zeta potentials of MC_20_, MC_50,_ and MC_100_ were −2.98, −5.90, and −9.88 mV, respectively and those of the MC_20_LA, MC_50_LA, and MC_100_LA micelles were −7.23, −16.57, and −18.38 mV, respectively (Figure 4). These results demonstrated that the micelles with and without LA were negatively charged and that LA can decrease the zeta-potential of the micelles. Because the p*K*_a_ of free fatty acids is approximately 5, in near physiological medium, the carboxyl group of the free fatty acids (LA in this study) will thus deprotonate and distribute to the negatively charged of micelles at pH = 7.4 [3,19]. Notably, LA, which is amphiphilic in its chemical structure, is not only solubilized in the core of micelles but also located at the interface of the core-shell of the micelle. Therefore, the presence of LA results in a reduction of the zeta potential [36].

#### 3.2.3. Transmission Electron Microscopy of the Micelles

**Figure 5:**
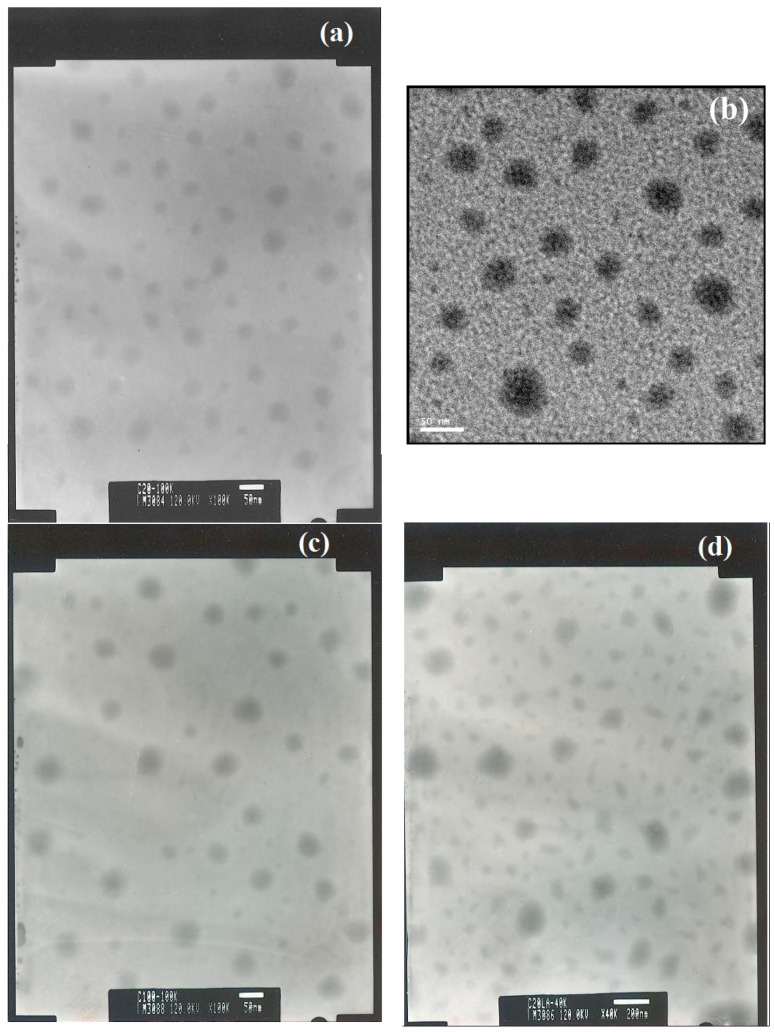

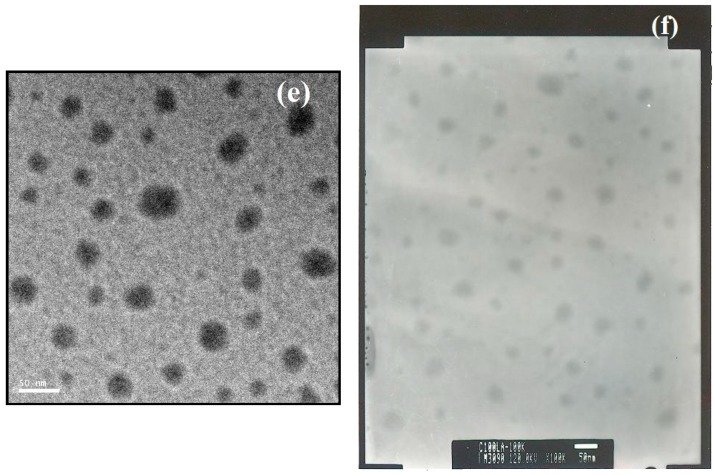
Transmission electron microscopy (TEM) images for the micelles without LA (**a**–**c**): MC_20_, MC_50,_ and MC_100_, respectively; the micelles loaded with LA (**d**–**f**): MC_20_LA, MC_50_LA, and MC_100_LA, respectively. The scale bar is 50 nm except for Figure 5d in which it is 200 nm.

Due to the nature of amphiphilic molecules, PCEC copolymers form a core-shell structure. TEM images of the blank micelles and LA-loaded micelles are shown in Figure 5. For blank micelles, the morphology of the dried micelles was spherical with diameters of 25–40 nm for MC_20_ (Figure 5a), 30–40 nm for MC_50_ (Figure 5b), and 40–50 nm for MC_100_ (Figure 5c). Because LA is a type of surfactant, we speculated that LA can form micelles (nanoparticles) under the same preparation protocol when no PCECs were added. However, there were no particles observed in the TEM images (images not shown). The diameter of the micelles was reduced to 20–30 nm for MC_50_LA (Figure 5e) and 20–40 nm for MC_100_LA (Figure 5f). The decreased micellar diameter after loading LA was appropriate with previous hypotheses [30], which demonstrated that reagents (LA in this study) could be incorporated into micelles.

Regarding the morphology of the dried micelles observed under TEM, there were small and non-spherical particles in the micelles after loading the LA, particularly in the MC_20_LA micelles (Figure 5d). When LA was loaded into the micelles, it interacted with the PCL segment through hydrophobic interactions, and the polar head group (carboxylic acid) may have been inserted into the shell layer. As a result, the nanoparticles can lose their spherical shape and/or adopt different morphologies [36]. Above a certain concentration known as the critical aggregation concentration, the micelles can self-assemble into different structures, e.g., rods, disks, spheres, bilayers or vesicles.

### 3.3. Drug Loading Content of LA in Micelle

The loading content of LA in LA-loaded micelles is shown in Table 4. LA encapsulated in the micelles was determined by HPLC after being derived from lauric acid naphthacyl ester, which was detected by fluorescent detector of HPLC. As shown in Table 4, the micelle concentration was obtained by freeze drying. After that, the as-prepared micelles were weighed to calculate the yield of preparation. It reflects the yield of the preparation method of micelles. One can observe that the concentration of micelles for MC_100_LA was one order of magnitude lower than that for the other two micelles. This could be attributed to the preparation of the micelles and the solubility of LA in the polymer segment of PCL [19]. Compared with dialysis, the thin-film hydration and ultra-sonication used in the present study can obtain micelles in a short period of time. PCL also has been known as a semi-crystalline polymer. The increased molecular weight of PCL in PCECs led to increased core crystallinity. As a result, the solubility of LA in that micelle was reduced [37]. In Table 4, the DLCs for MC_20_LA, MC_50_LA, and MC_100_LA were 3.27%, 9.75%, and 15.42%, respectively. Apparently, the DLCs were dependent on the composition of PCL-PEG-PCL copolymers. The LA was physically encapsulated into the micelles due to the hydrophobic interactions between LA and the core segment of PCL. Hence, the DLC was affected by the interactions between LA and PCL, the crystallinity of PCL and the hydrogen bond interactions [34]. In our study, the favorable interactions between PCL and LA were enhanced in the cores of the micelles, leading to an increase in the DLC of LA. Furthermore, MC_50_LA prepared with 10 mg of PCL-PEG-PCL and 2 mg of LA reached the highest concentration of encapsulated LA (e.g., 323.75 µg/mL), which required larger space to accommodate LA than did the other two micelles. Therefore, the particle size of MC_50_ after loading LA did not apparently change compared with those of MC_20_ and MC_100_.

### 3.4. Minimum Inhibitory Concentration

**Figure 6:**
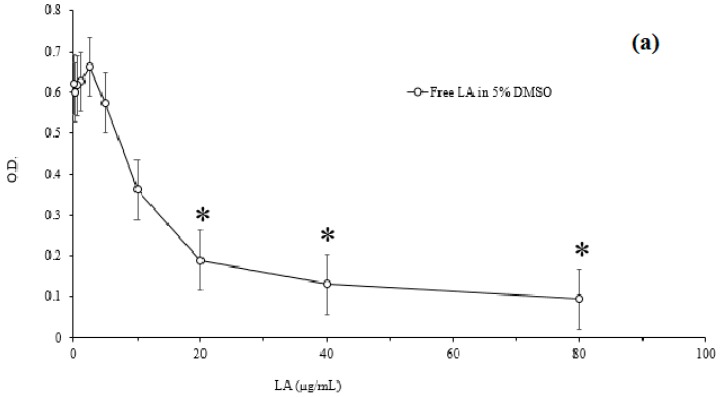

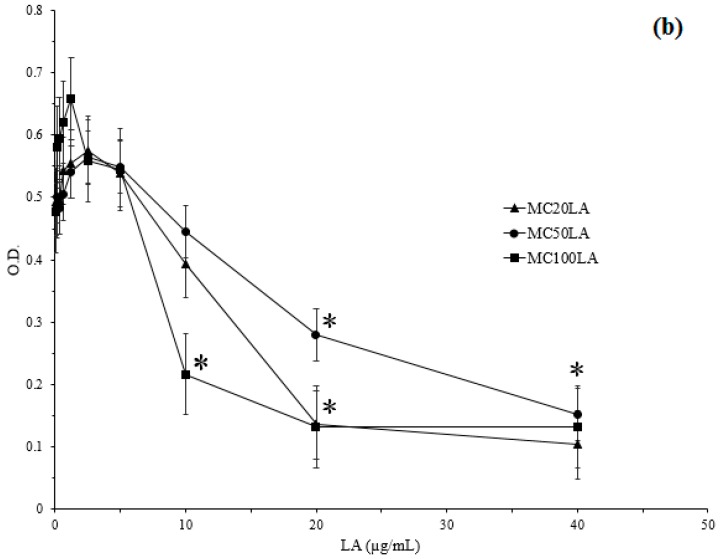
The minimum inhibitory concentration (MIC) assay. (**a**) MIC of free LA (in 5% DMSO); (**b**) MICs of MC_20_LA, MC_50_LA, and MC_10_LA. The initial concentration of bacteria was 10^6^ CFU/mL; the optical density of bacteria at 600 nm was measured by ELISA. * indicates significant difference compared with a PBS control by student’s *t*-test (*n* = 3).

Figure 6 represents the MICs of free LA and various LA-loaded micelles. The MICs of LA-loaded micelles were compared with those of free LA to evaluate the effect of LA on the inhibition of *P. acnes* growth. As shown in Figure 6, the lowest concentration to prevent bacterial growth in free LA was 20 μg/mL, which was lower than that of BPO (100 μg/mL) [38]. The same value of MIC was found for both MC_20_LA and MC_50_LA. This result proved that LA is a promising antibacterial to inhibit the growth of *P. acnes*. The DLCs for the three micelles were closely related to the MICs. The bacteria were cultured in the liquid medium to determine the MICs. We found that the bacteriostatic action was significant for MC_100_LA, whereas MC_20_LA and MC_50_LA did not effectively inhibit the growth of *P. acnes*. Notably, only 10 μg/mL MC_100_LA was required to achieve the same inhibitory effect of free LA. This was because the drug loading content was higher, leading to an improved antibacterial effect. According to the literature, the water solubility of LA is 4.81 µg/mL [39], and thus the payloads of the PCEC micelles were 35.8, 67.3, and 21.9-fold for MC_20_LA, MC_50_LA, and MC_10_LA, respectively, relative to free LA in water. These data showed the advantage of the particulate-based drug delivery systems to increase the solubility of water-insoluble drugs.

### 3.5. The Minimum Bactericidal Concentration

**Figure 7:**
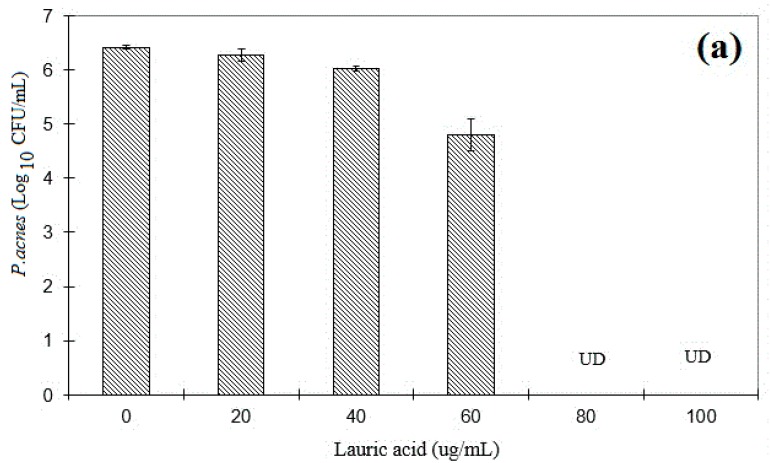

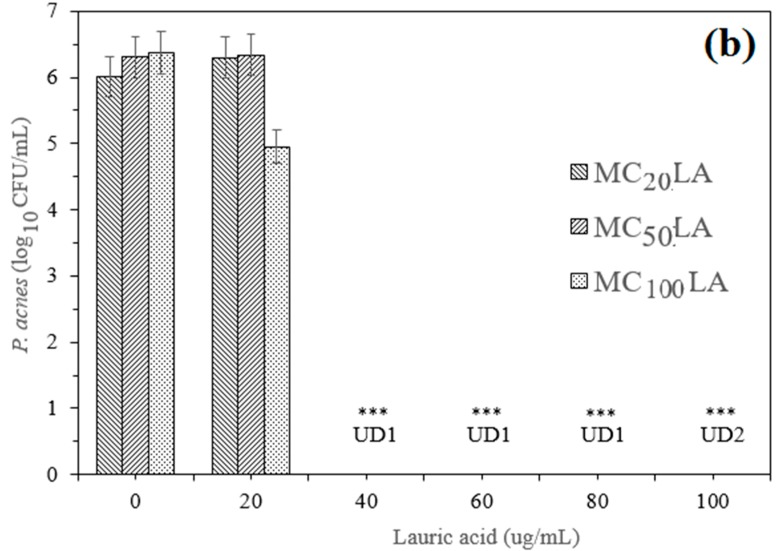
The minimum bactericidal concentration (MBC) assay. UD: no colonies on a Noble agar plate. UD1: there were no colonies for MC_20_LA, MC_50_LA, and MC_100_LA. UD2: there were no colonies for MC_20_LA and MC_50_LA; 100 µg/mL MC_100_LA was not tested; (**a**) LA dissolved in 5% DMSO or (**b**) LA-loaded micelles co-cultured with 10^7^ CFU/mL of *P. acnes* in reinforced clostridial medium (RCM) medium for 5 h and then spotted on Noble agar plate for 96 h, followed by colony counting; *** indicates significant difference compared to blank micelles made of respective copolymer by student’s *t*-test (*n* = 3).

**Figure 8:**
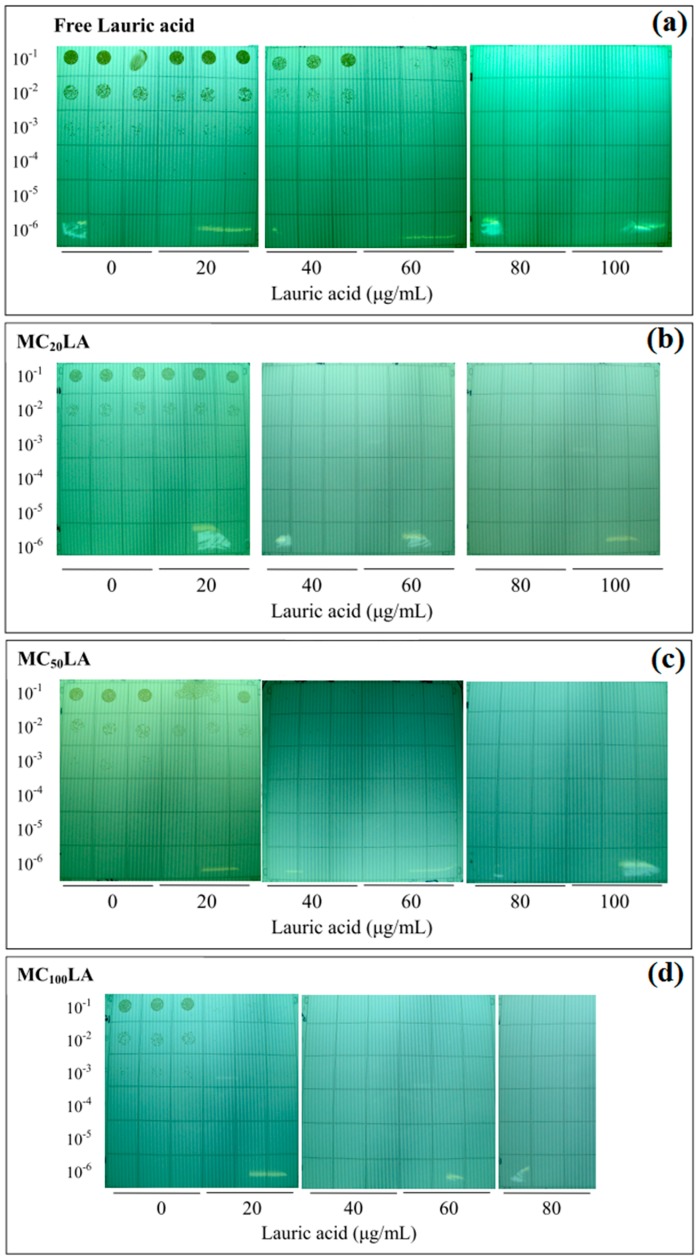
*P. acnes* grown on RCM in the presence of free LA (**a**) and LA-loaded micelles (**b**–**d**). The images were photographed with a digital camera. For each type of micelle, three columns (*n* = 3) of the bacterial colony were tested. The *Y* axis indicates the concentration of *P. acnes.*

*P. acnes* were diluted with PBS and spotted on Noble agar plates after incubation to determine the CFUs. It has been found that when the concentration of free LA was higher than 80 μg/mL (Figure 7a), over 99.9% of *P. acnes* were killed. This value was the same as previous studies [19]. The MBCs of MC_20_LA, MC_50_LA, and MC_100_LA were all 40 μg/mL (Figure 7b), which revealed that LA-loaded micelles produce many potent bactericidal effects than does free LA on *P. acnes*. This was due to over 20-fold more LA loaded onto the micelles compared with free LA in PBS [3]. These results again displayed the advantage of the high payload of LA in the micelles when co-cultured with *P. acnes.* Figure 8 displays the colonies of *P. acnes* grown on RCM when co-cultured with the PCEC micelles. It was observed that 20 μg/mL MC_100_LA could partially kill bacteria, whereas the other micelles at the same concentration remained non-effective (Figure 8d). This showed the relatively stronger antibacterial effect among the other micelle formulations. This performance was also observed via the MIC experiment in Section 3.4. This could be explained because MC_100_LA was characterized as the highest loading content. Furthermore, all of the micellar formulations carried a lethal dosage of LA against *P. acnes*.

Since LA was found to inhibit the growth of skin commensal *P. acnes* in 2009 [3], various dosage forms have been developed. For example, Silva et al. reported solid lipid nanoparticles containing retinoic acid and LA for the topical treatment of acne [40]. Bai and Hsueh used tea tree essential oil encapsulated in silk fibroin/polyvinyl alcohol membrane to manage the inflammation and acne in *P. acnes*-infected mouse ears [41]. Huang et al. reported that LA not only inhibited the growth of *P. acnes* but also reduced the inflammation reaction in mice via the NF-κB and MAP kinase pathways [2]. Overall, the use of free fatty acids in the management of acne vulgaris is a potential approach. A recent study in the skin microbiome revealed that *Staphylococcus epidermidis* can limit the growth of *P. acnes* via fermentation products, such as free fatty acids [42]. This indicated that our finding in the effect of micellar LA on killing *P. acnes* is part of the skin microbiome and the engineered nanoparticles; the micellar nanoparticles in this study can exert higher potency of LA toward the bactericidal effect on *P. acnes*, compared with free LA.

## 4. Conclusions

DMSO has been reported to solubilize water-insoluble LA in the treatment of acne vulgaris. It is known that acne is induced by the overgrowth of the skin bacterium *P. acnes*. However, DMSO can induce toxic side effects in skin cells. In this study, the amphiphilic block copolymer PCEC with different molecular weights was successfully synthesized, and LA was loaded in the PCEC micelles using the hydration method. The particle size analysis and zeta potential displayed that LA can be encapsulated in the micellar nanoparticles, and the dosage form of LA-loaded PCEC micelles can replace the use of DMSO as the excipient of LA in the treatment of skin disorders. Moreover, among various LA-loaded micellar nanoparticles, the minimal inhibitory concentration and minimal bactericidal concentration of LA-loaded micelles made of longer hydrophobic PCL segment led to a higher LA payload and exerted the strongest antibacterial effects. Therefore, the promising results of this study merit further study of the efficacy of LA against *P. acnes* infection in future animal studies.

## Figures and Tables

**Table 1:** Preparation of blank nano-sized micelles and lauric acid (LA)-loaded micelles with different polymer to LA ratios. The copolymer name subscript indicates 1/100 of the designed molecular weights of each segment.

PCEC copolymer	PCEC:LA (mg:mg)	Abbreviation
PC_20_E_40_C_20_	10:0	MC_20_
	10:2	MC_20_LA
PC_50_E_40_C_50_	10:0	MC_50_
	10:2	MC_50_LA
PC_100_E_40_C_100_	10:0	MC_100_
	10:2	MC_100_LA

**Table 2:** Molecular weights of PCECs and critical micelle concentration (CMC). The first column displays the theoretical molecular weights of the PCECs. All molecular weights are expressed asg/mol.

		${\bar{M}}_{NMR\;{}^{a}}$	${\bar{M}}_{n\;{}^{b}}$	${\bar{M}}_{w\;{}^{b}}$	Polydispersity index	CMC (wt %)
PC_20_E_40_C_20_	8000	8127	8320	9727	1.17	5.43 × 10^−3^
PC_50_E_40_C_50_	14,000	13,698	10,831	13,999	1.29	4.17 × 10^−3^
PC_100_E_40_C_100_	24,000	22,571	14,154	18,838	1.33	2.16 × 10^−3^

^a^: ${\bar{M}}_{\text{MNR}}$ obtained from the ^1^H Nuclear Magnetic Resonance spectroscopy (^1^H NMR) analysis; ^b^: ${\bar{M}}_{n}\text{ and }{\bar{M}}_{w}$ obtained from Gel Permeation Chromatography (GPC) analysis.

**Table 3:** The particle sizes and distribution of the micelles. PDI stands for polydispersity index.

Micelle	PCEC:LA (mg/mg)	Average particle size (nm)	PDI	Zeta potential (mV)
MC_20_	10:0	50	0.27	−2.98
MC_20_LA	10:2	27	0.33	−7.23
MC_50_	10:0	78	0.24	−5.90
MC_50_LA	10:2	68	0.21	−16.57
MC_100_	10:0	198	0.14	−9.88
MC_100_LA	10:2	89	0.21	−18.38

**Table 4:** Concentration of LA loaded micelles and drug loading content.

Micelle	Concentration of LA in micelles ^a^ (µg/mL)	Concentration of micelles ^b^ (mg/mL)	Drug loading content ^c^ (%)
MC_20_LA	172.24 ± 6.19	5.27 ± 0.06	3.27 ± 0.15
MC_50_LA	323.75 ± 15.33	3.43 ± 0.7	9.75 ± 2.35
MC_100_LA	105.30 ± 12.56	0.7 ± 0.1	15.42 ± 4.02

^a^ Concentration of encapsulated LA measured by reversed-phase high performance liquid chromatography (HPLC); ^b^ as-prepared micelles containing the polymer and LA were freeze-dried and weighed; ^c^ the drug loading content calculated by equation: $\frac{\text{amount of encapsulated LA}}{\text{amount of polymer}+\text{LA}}\times 100\%.$

## Supplementary Materials

Supplementary Materials can be found at www.mdpi.com/2073-4360/8/9/321/s1.
