# Supplementary material for: Bactericidal Effect of Lauric Acid-Loaded PCL-PEG-PCL Nano-Sized Micelles on Skin Commensal Propionibacterium acnes
Source: Polymers (Basel). 2016 Aug 27;8(9):321. doi: 10.3390/polym8090321 (PMC6431869; doi:10.3390/polym8090321)
Supplement: Supplementary file 1 [file polymers-08-00321-s001.pdf]

Supplementary Materials: Bactericidal Effect of Lauric Acid-Loaded PCL-PEG-PCL Nano-Sized Micelles on Skin Commensal *Propionibacterium acnes*

Thi-Quynh-Mai Tran, Ming-Fa Hsieh, Keng-Lun Chang, Quoc-Hue Pho, Van-Cuong Nguyen, Ching-Yi Cheng and Chun-Ming Huang

1. Supplements of Triblock Copolymer: ^{1}H NMR Spectra and DSC Thermograms

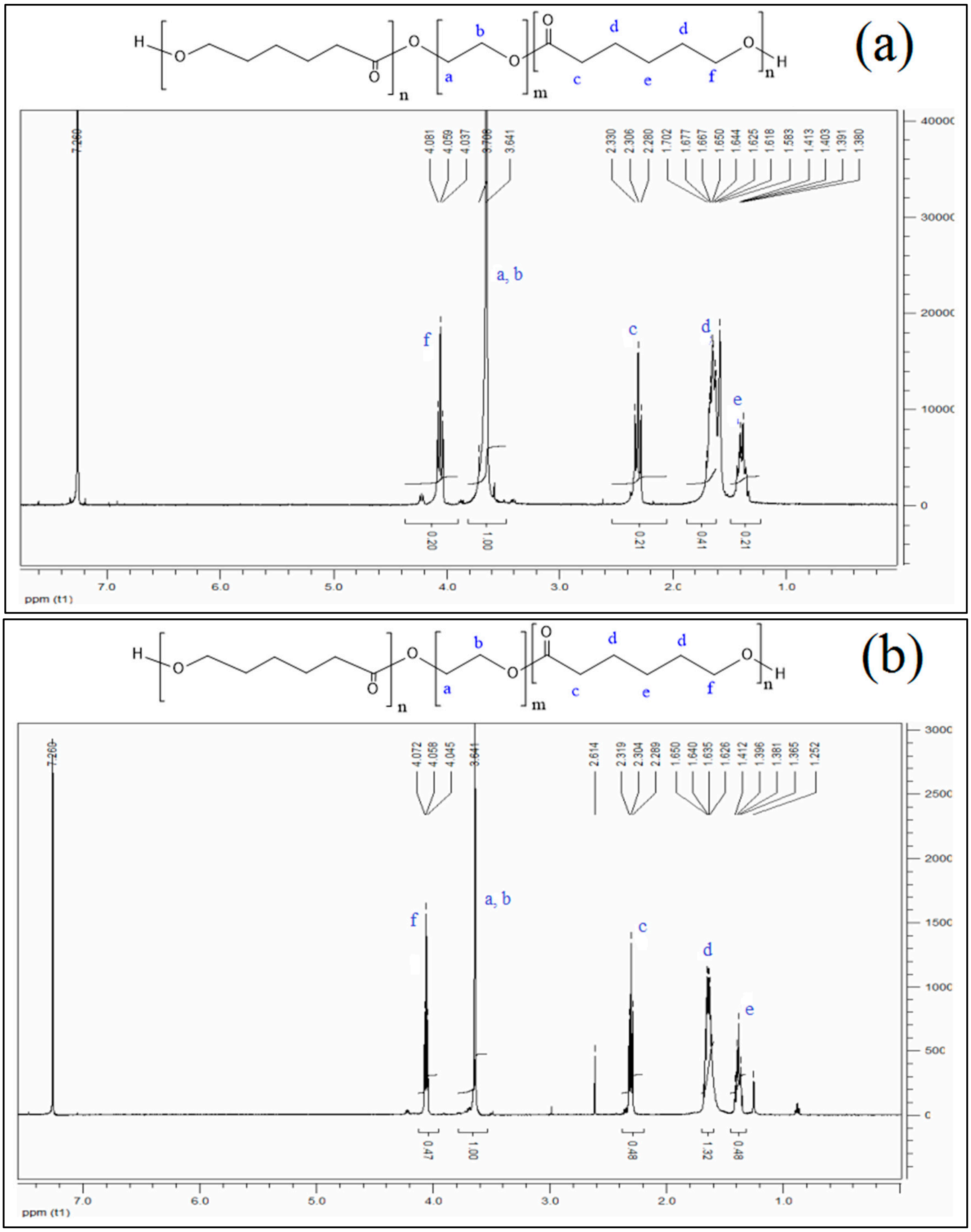

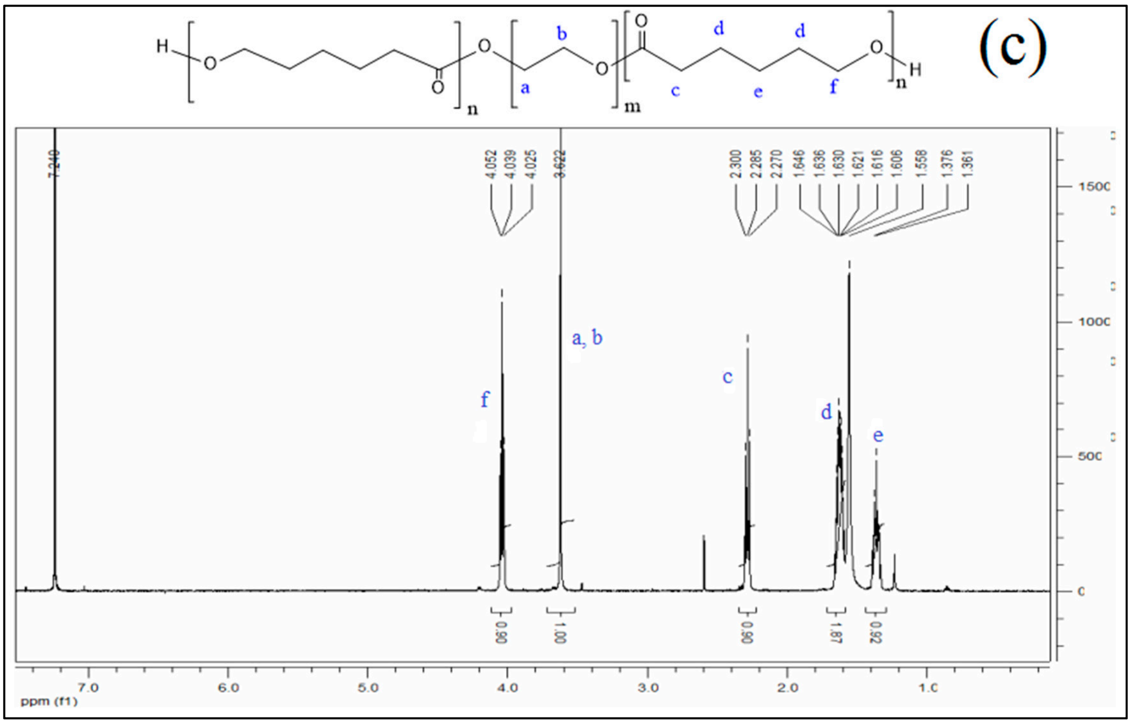

Figure S1. The ^{1}H NMR spectra of triblock copolymers: (**a**) $PC_{20}E_{40}C_{20}$; (**b**) $PC_{50}E_{40}C_{50}$; (**c**) $PC_{100}E_{40}C_{100}$.

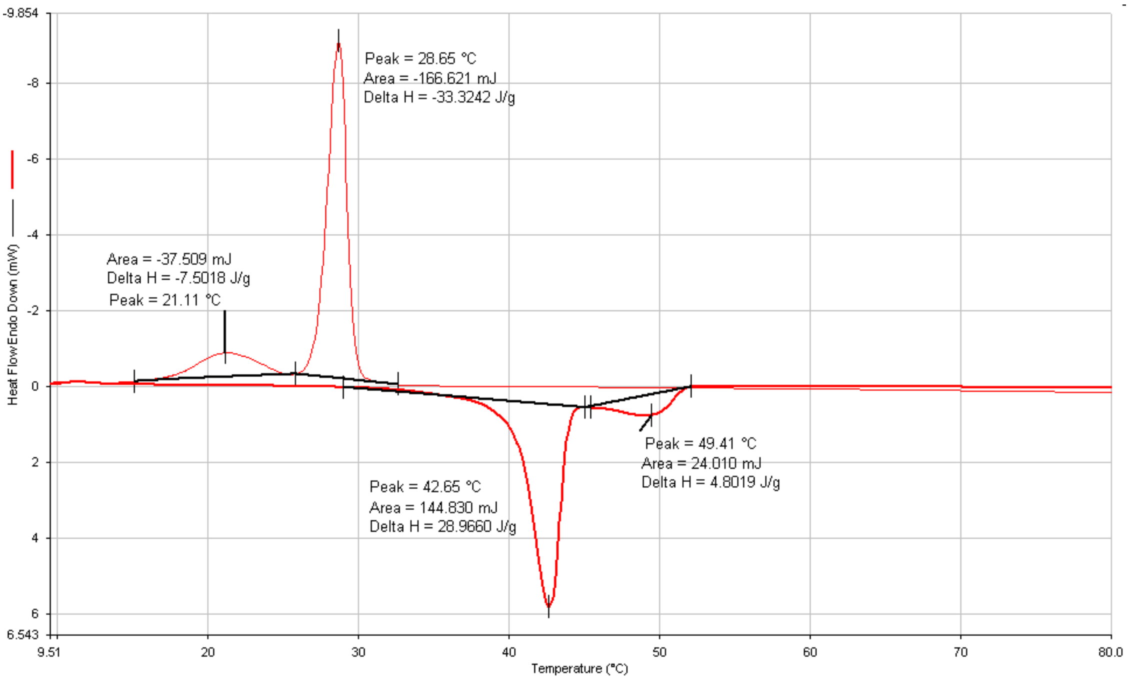

(**a**)

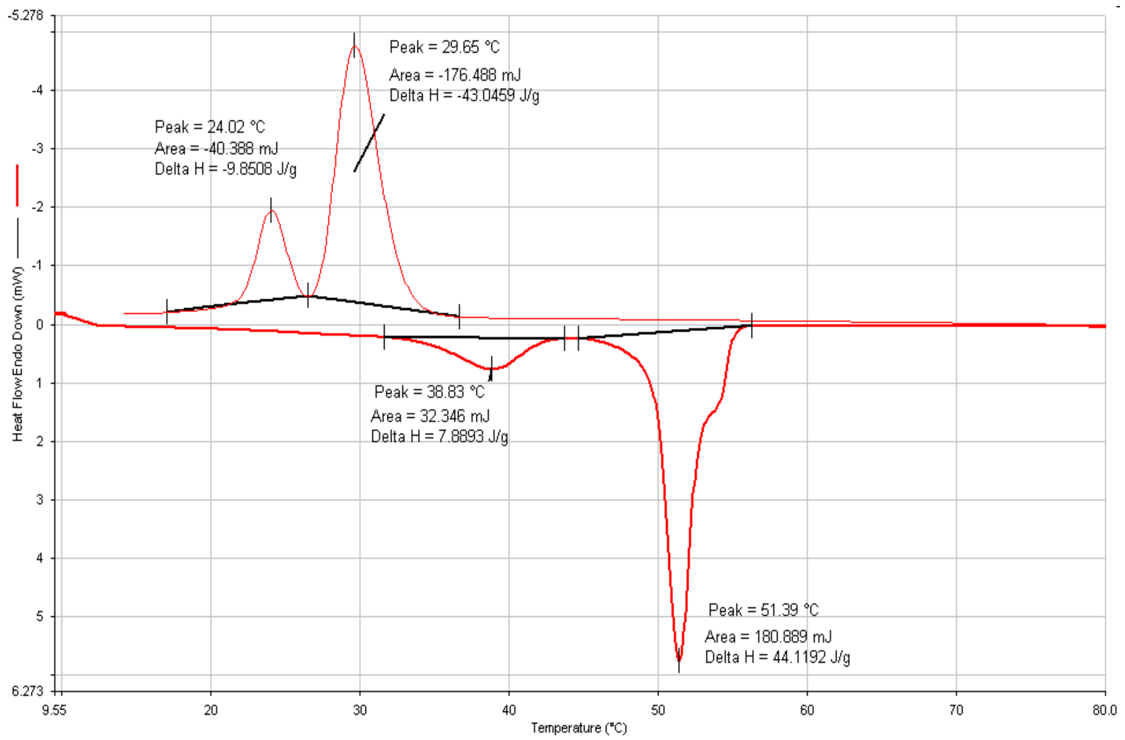

(**b**)

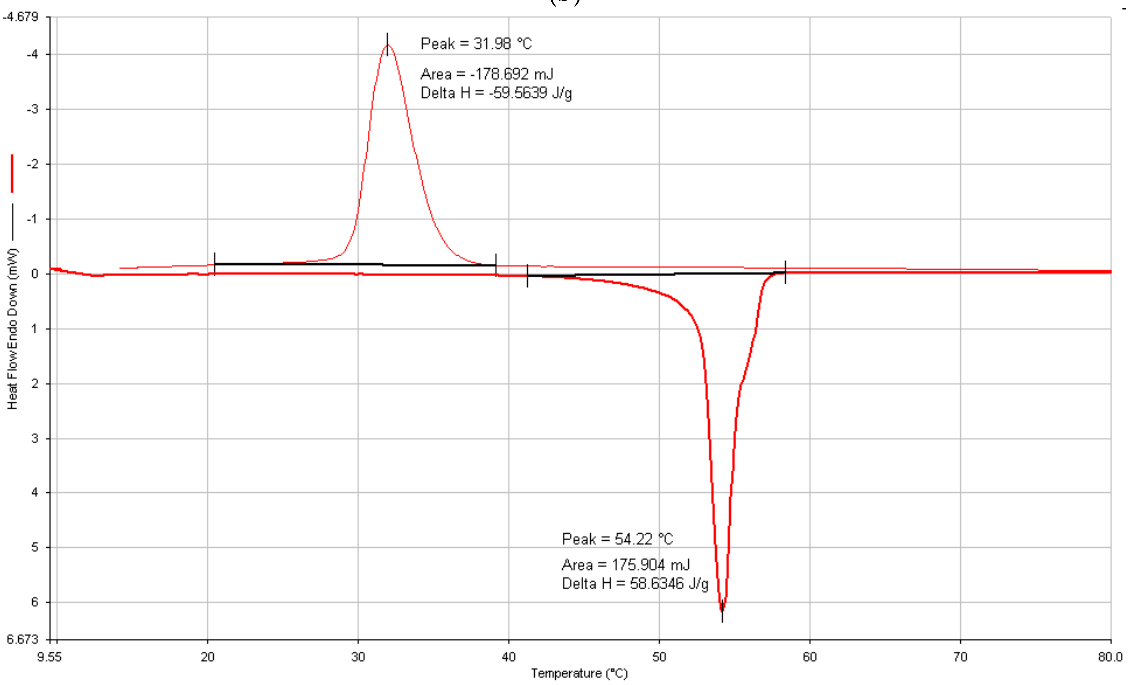

(**c**)

Figure S2. DSC thermograms of triblock copolymers: (**a**) $PC_{20}E_{40}C_{20}$; (**b**) $PC_{50}E_{40}C_{50}$ and (**c**) $PC_{100}E_{40}C_{100}$. The upper half of the thermogram represents the second cooling curve and the other displayed the second heating curve.

2. Preparation and Characterization of Diblock PEG-PCL Copolymer

In this section, mPEG-PCL diblock copolymers were synthesized by ring-opening polymerization reaction using $Sn(Oct)_2$ as a catalyst and mPEG (5000 Da) as an initiator. Theoretical molecular weights of PCL segment were designed to be 7500, 10000, 15000 Da corresponding to PC_{75}, PC_{100} and PC_{150}, respectively. The reaction was carried out at 130 °C for 5 h. Moreover, the ratios of hydrophilic segment: hydrophobic segment were 1:0.75, 1:1, 1:2, respectively. The physicochemical properties of copolymers were characterized by ^1H NMR, FTIR and DSC, respectively. Lastly, the

micelles were prepared and characterized by particle size analyzer. These results were used to compare to that of triblock copolymers PCL-PEG-PCL.

3. Characterization of mPEG-PCL Diblock Copolymers

3.1. Critical Micelle Concentration of the Micelles

Regarding to the pyrene 1:3 ratio method, CMCs of copolymer were determined based on the relationship of I_1/I_3 intensity ratio of pyrene included in micelles and concentrations. As shown in Figure S7, the CMC was determined at the center point of the sigmoid. It was found that the CMCs of PC_{75}, PC_{100}, PC_{150} (shown in Table S1) were 16.4×10^{-3}, 8.91×10^{-3} and 4.47×10^{-3} wt %, respectively. Apparently the CMCs reduced from 16.4×10^{-3} to 4.47×10^{-3} (wt %) when the molecular weight (chain length) of the hydrophobic PCL segment increased from 2500 Da to 10,000 Da. The values of polymeric micelles mainly depend on the hydrophobic segment of copolymers. The comparison of CMC of diblock and triblock copolymer is described in details in Section 3.2.1 of the main text.

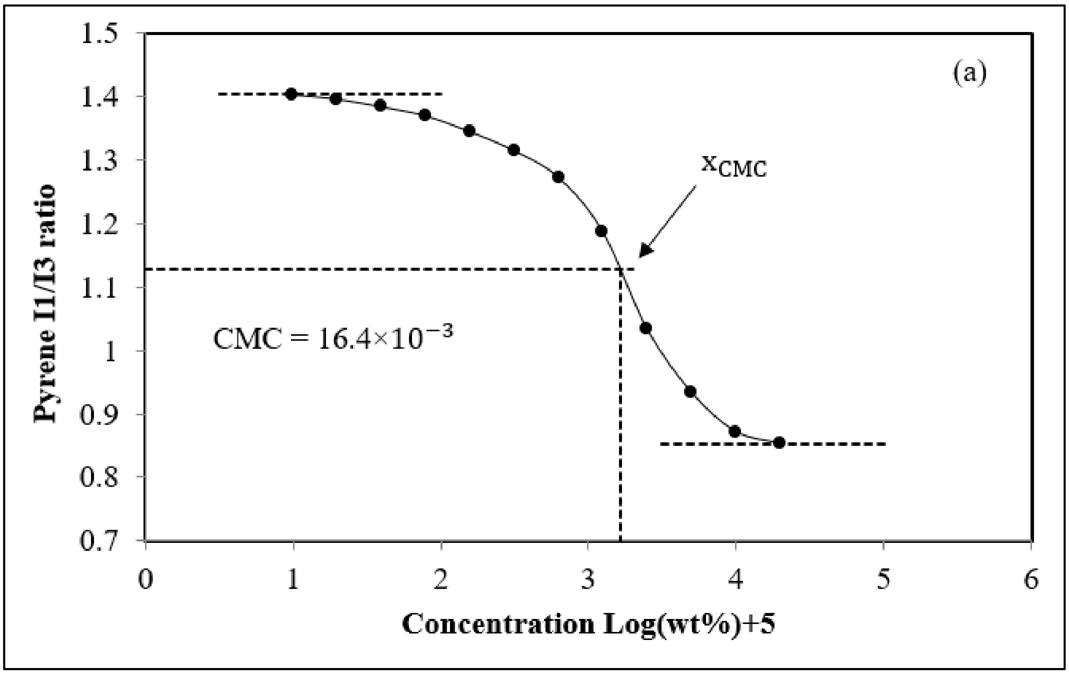

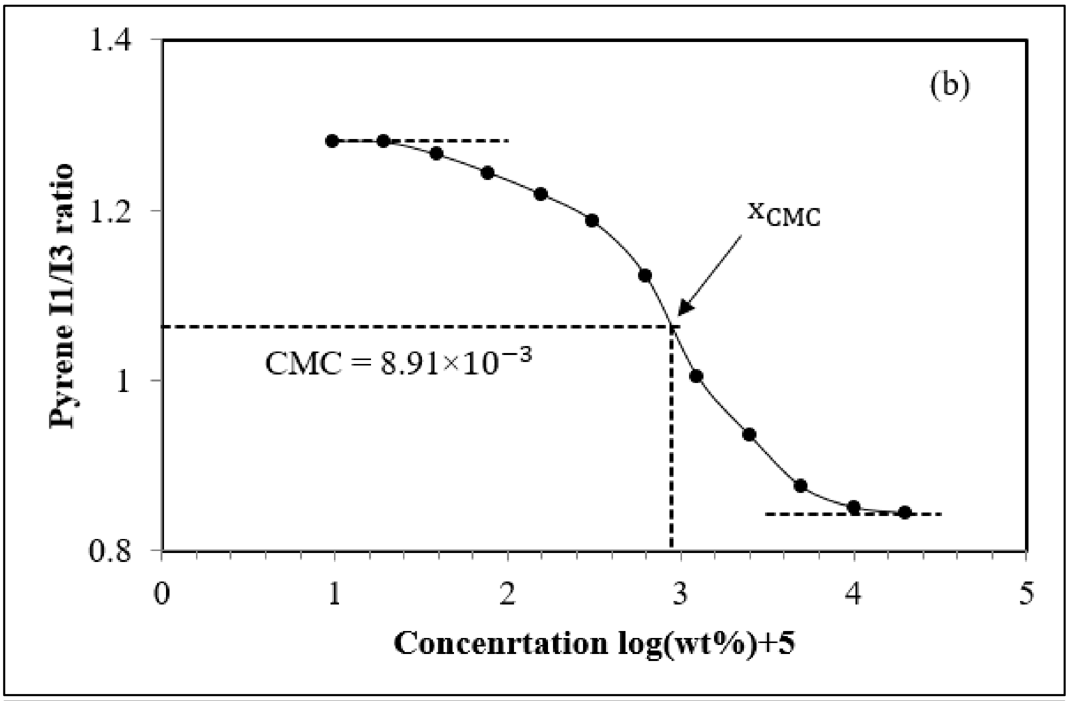

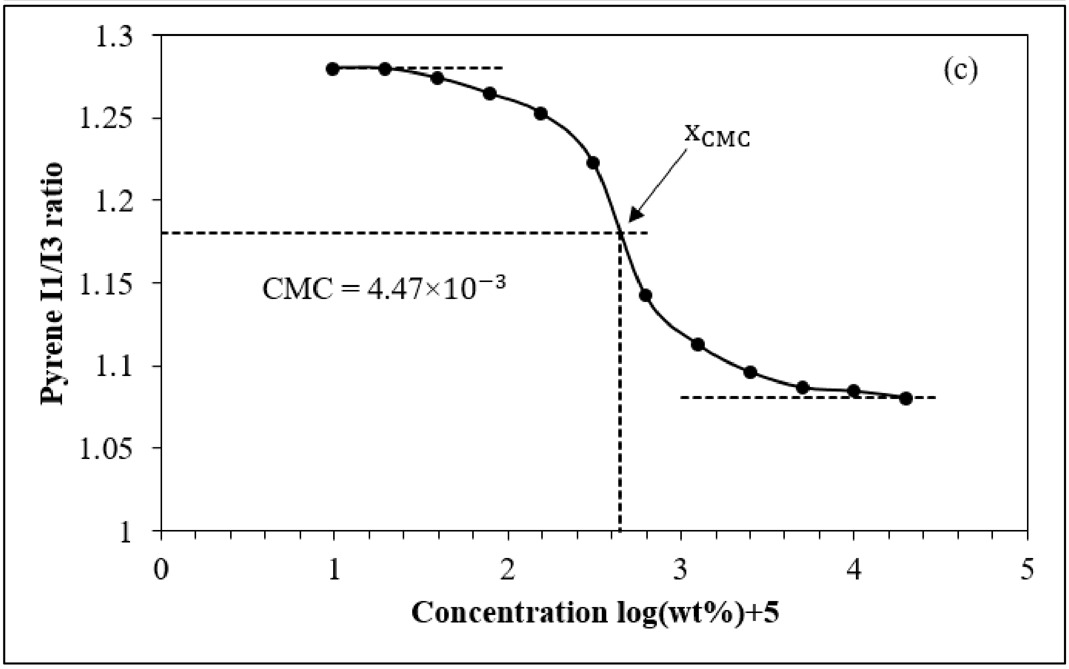

Figure S3. Measurement of CMC values for (**a**) PC_{75}; (**b**) PC_{100} and (**c**) PC_{150}.

3.2. ^{1}H NMR and FT-IR Characterization of Molecular Structure of Diblock Copolymers

mPEG-PCL diblock copolymers were synthesized using the ring-opening polymerization of ε-CL in the presence of mPEG. The hydroxyl end group initiated the ring opening of ε-CL. The Chemical structure of mPEG-PCL diblock copolymers was determined by ^{1}H NMR in $CDCl_3$. The presence of CH_2 group in PCL was observed around 1.3 ppm, 1.6 ppm, 2.3 ppm and 4 ppm as shown in Figure S4. The methoxy protons (OCH_3 group) of mPEG was observed at 3.4 ppm and the peak at 3.64 ppm was assigned as the methylene protons (CH_2 group) of mPEG. Table S1 summarized the characteristics of synthesized diblock copolymers.

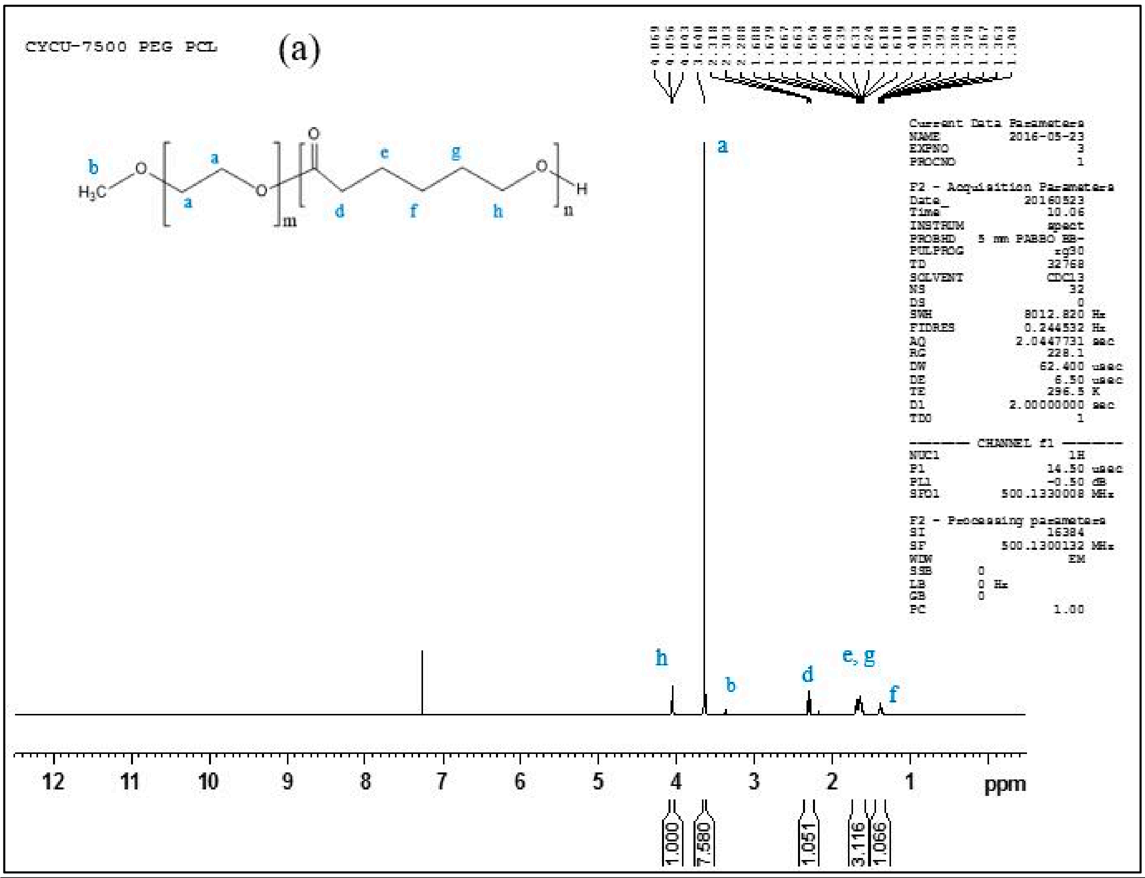
CYCU-7500 PEG PCL
(a)
Current Data Parameters
NAME 2016-05-23
EXPNO 3
PROCNO 1
F2 - Acquisition Parameters
Date_ 20160523
Time 10.06
INSTRUM spect
PROBHD 5 mm PABBO BB-
PULPROG zg30
TD 32768
SOLVENT CDCl3
NS 32
DS 0
SWH 8012.820 Hz
FIDRES 0.244532 Hz
AQ 2.0447731 sec
RG 228.1
DW 62.400 usec
DE 6.50 usec
TE 296.5 K
D1 2.00000000 sec
TD0 1
CHANNEL f1
NUC1 1H
P1 14.50 usec
PL1 -0.50 dB
SFO1 500.1330008 MHz
F2 - Processing parameters
SI 16384
SF 500.1300132 MHz
WDW EM
SSB 0
LB 0 Hz
GB 0
PC 1.00
a
h
b
d
e, g
f
ppm
1.000
7.580
1.051
3.116
1.066

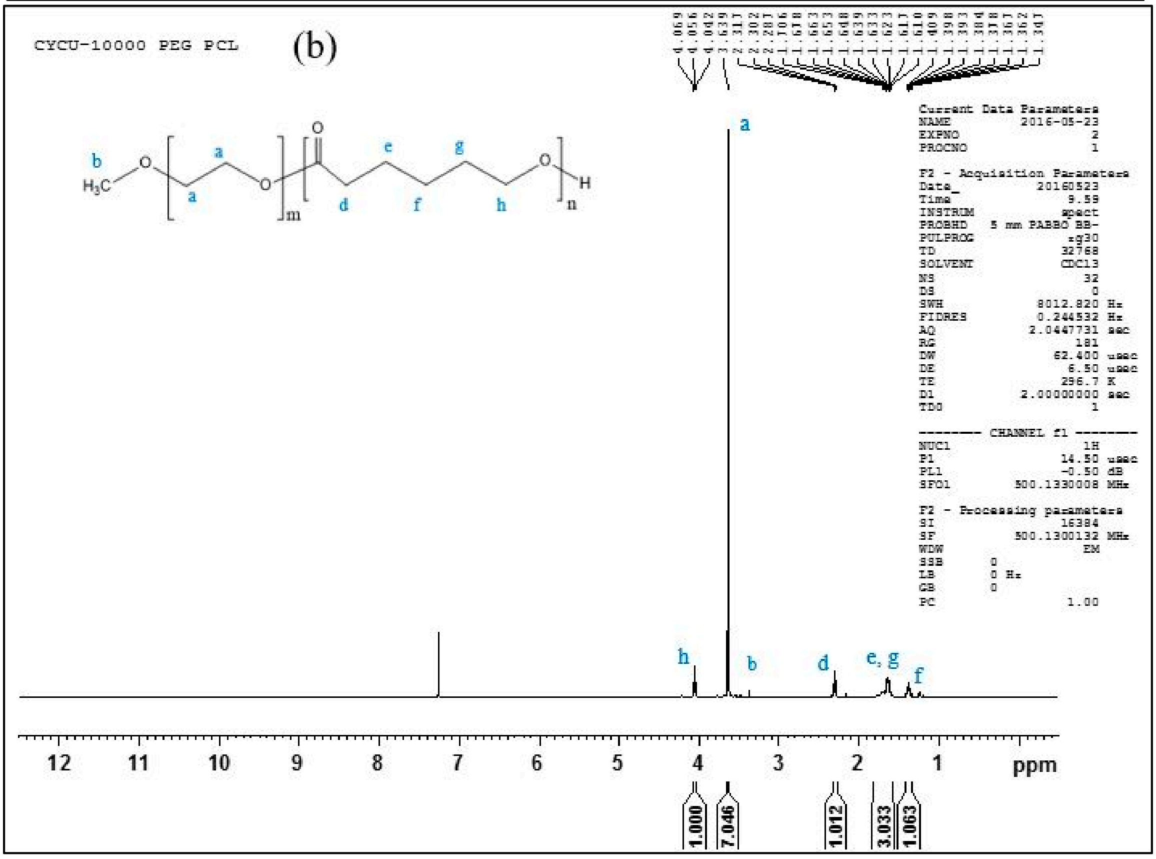
CYCU-10000 PEG PCL
(b)
Current Data Parameters
NAME 2016-05-23
EXPNO 2
PROCNO 1
F2 - Acquisition Parameters
Date_ 20160523
Time 9.59
INSTRUM spect
PROBHD 5 mm PABBO BB-
PULPROG zg30
TD 32768
SOLVENT CDCl3
NS 32
DS 0
SWH 8012.820 Hz
FIDRES 0.244532 Hz
AQ 2.0447731 sec
RG 181
DW 62.400 usec
DE 6.50 usec
TE 296.7 K
D1 2.00000000 sec
TD0 1
CHANNEL f1
NUC1 1H
P1 14.50 usec
PL1 -0.50 dB
SFO1 500.1330008 MHz
F2 - Processing parameters
SI 16384
SF 500.1300132 MHz
WDW EM
SSB 0
LB 0 Hz
GB 0
PC 1.00
a
h
b
d
e, g
f
ppm
1.000
7.046
1.012
3.033
1.063

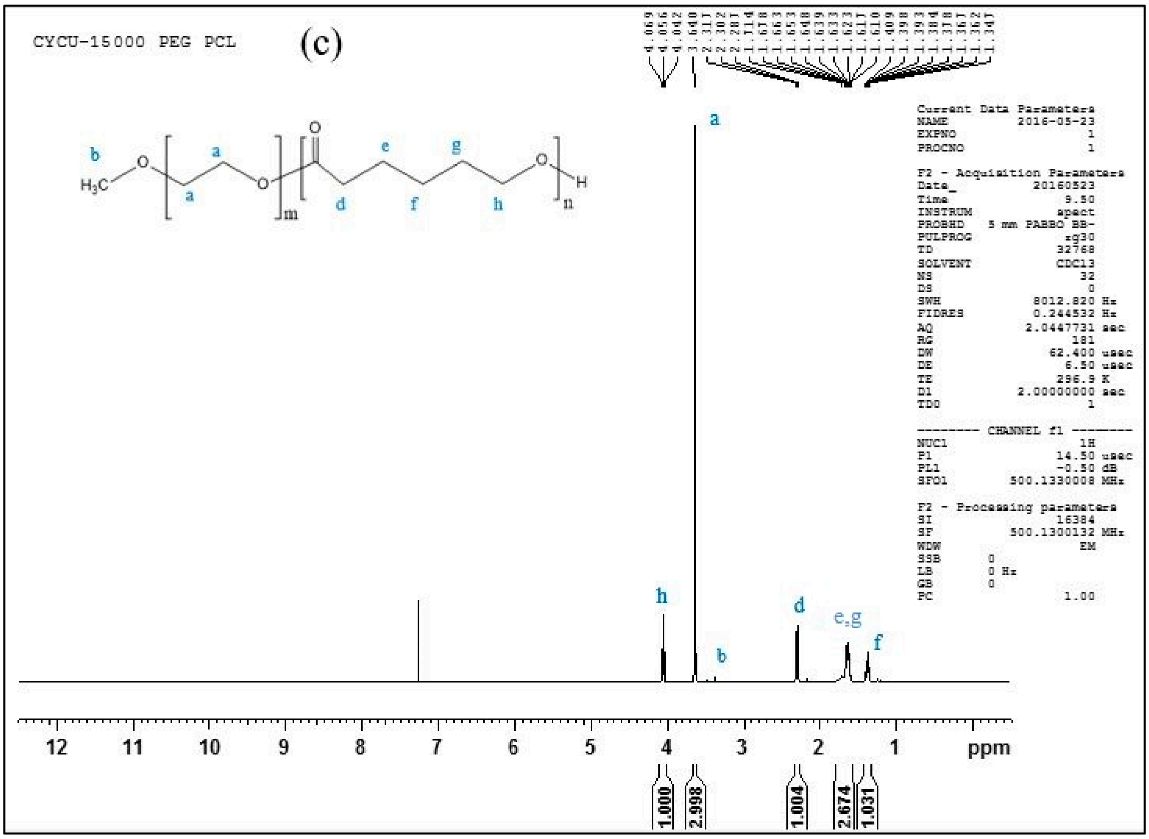

Figure S4. The ^{1}H-NMR spectra of diblock copolymers: PC_{75} (**a**); PC_{100} (**b**); PC_{150} (**c**).

Functional groups of mPEG-PCL diblock copolymers were characterized by FT-IR spectrophotometer. As one can see in Figure S5, all spectra show typical peaks of C–H stretching in PCL segment at 2890.7–2946.7 cm^{-1}. In addition, typical peaks of C=O groups in PCL segment appeared at 1722.8–1731.7 cm^{-1}. Lastly, a specific peak at 1110.8–1180.2 cm^{-1} indicated C–O–C stretching in PEG segment. Moreover, when increasing molecular weight of PCL segment in the diblock copolymer, the intensity of C=O of PCL became stronger.

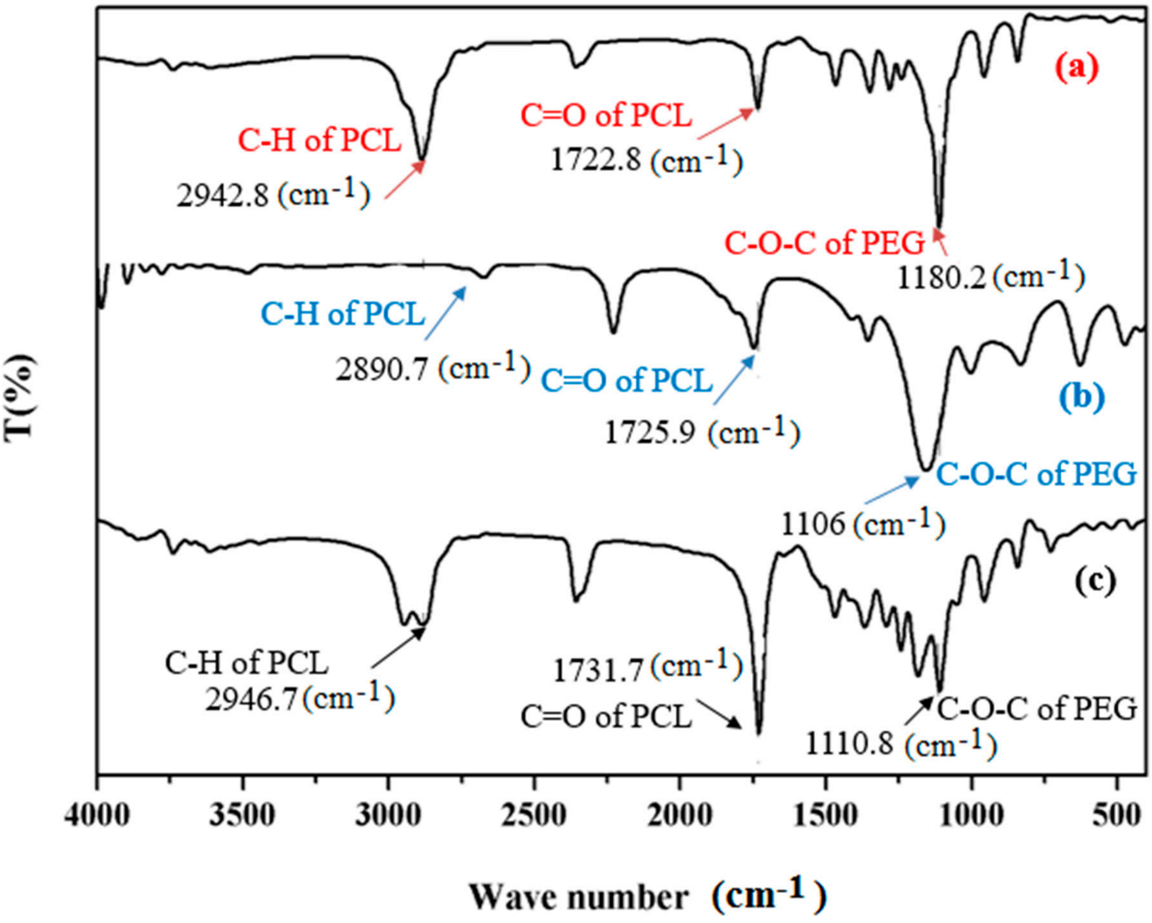

Figure S5. FT-IR spectra of diblock copolymers: (**a**) PC_{75}; (**b**) PC_{100} and (**c**) PC_{150}.

Table S1. Molecular characteristics of the synthesized diblock copolymers.

Copolymer	Feeding ratio of PEG/ε-CL	M_w [a] (Da)	M_w [b] (Da)	CMC (wt %)
PC_{75}	2	7,500	6959	16.4×10^{-3}
PC_{100}	1	10,000	9359	8.91×10^{-3}
PC_{150}	0.5	15,000	14,139	4.47×10^{-3}

[a]: the theoretical molecular weight of copolymer. [b]: the molecular weight of copolymer determined by ^{1}H NMR.

3.3. DSC Thermograms of Diblock Copolymers

As observed in Figure S6, the components of hydrophobic segment affected the melting point of copolymers. Particularly, the melting point decreased from 56.65 to 54.83 °C, when increasing molecular weight of PCL segment from 2500 to 10,000 Da.

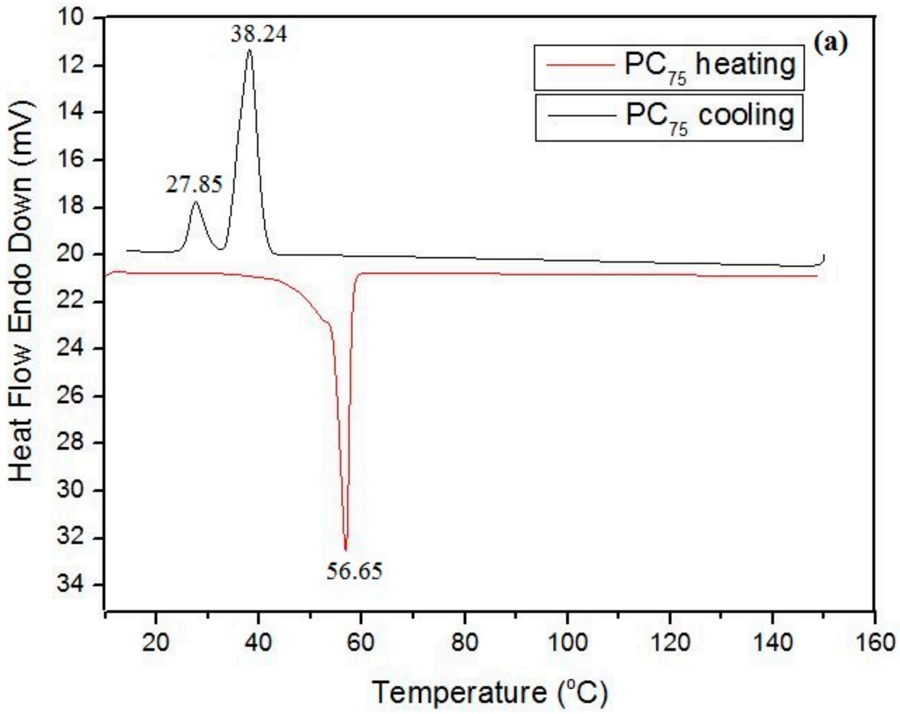
(a)
PC75 heating
PC75 cooling
38.24
27.85
56.65
Heat Flow Endo Down (mV)
Temperature (°C)

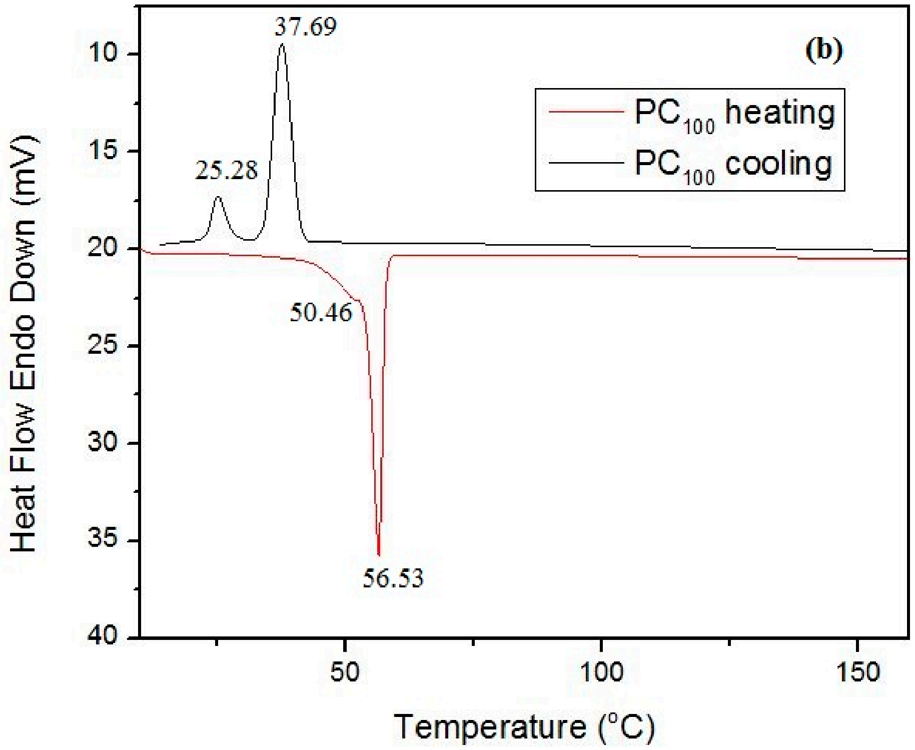
(b)
PC100 heating
PC100 cooling
37.69
25.28
50.46
56.53
Heat Flow Endo Down (mV)
Temperature (°C)

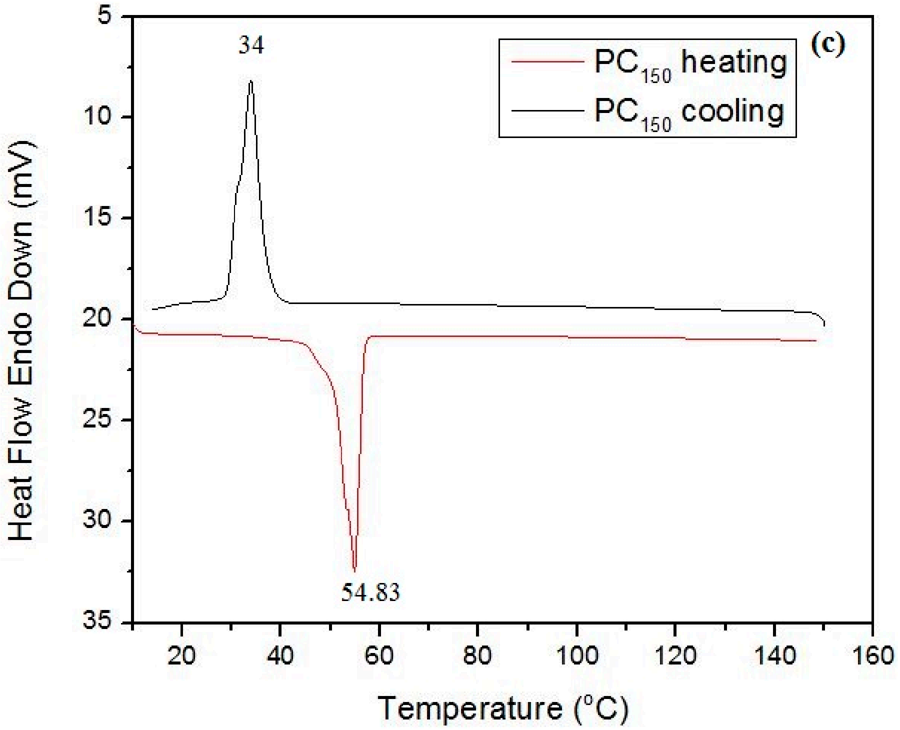

Figure S6. DSC Thermograms of PC_{75} (**a**); PC_{100} (**b**) and PC_{150} (**c**) diblock copolymers; the upper half side of the diagram represents for the second cooling curve and the other displays the second heating curve.

3.4. Particle Size and Distribution of the Micelles

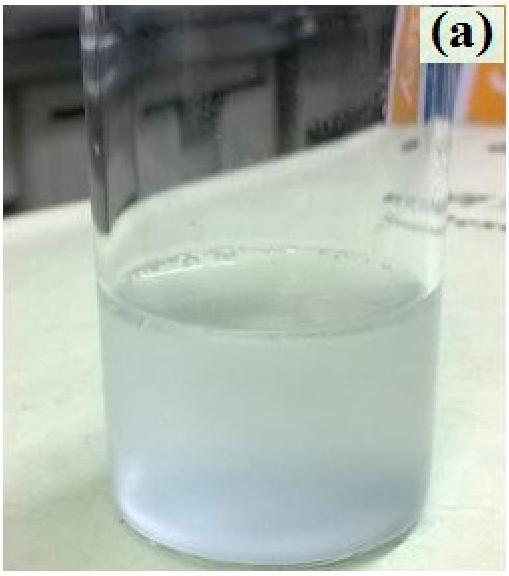

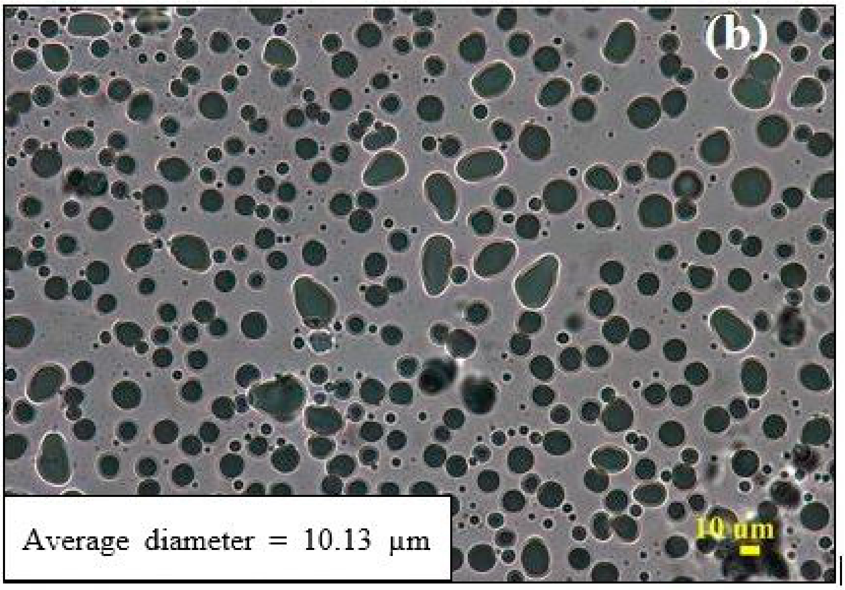
(b)
Average diameter = 10.13 μm
10 μm

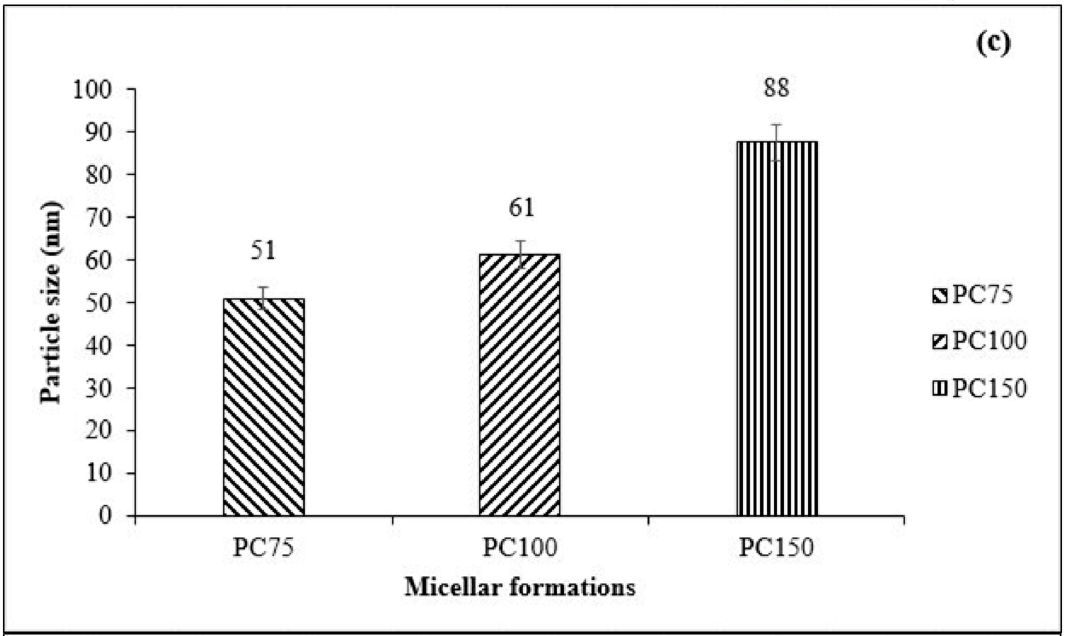
(c)
Particle size (nm)
100
90
80
70
60
50
40
30
20
10
0
51
61
88
PC75
PC100
PC150
Micellar formations
PC75
PC100
PC150

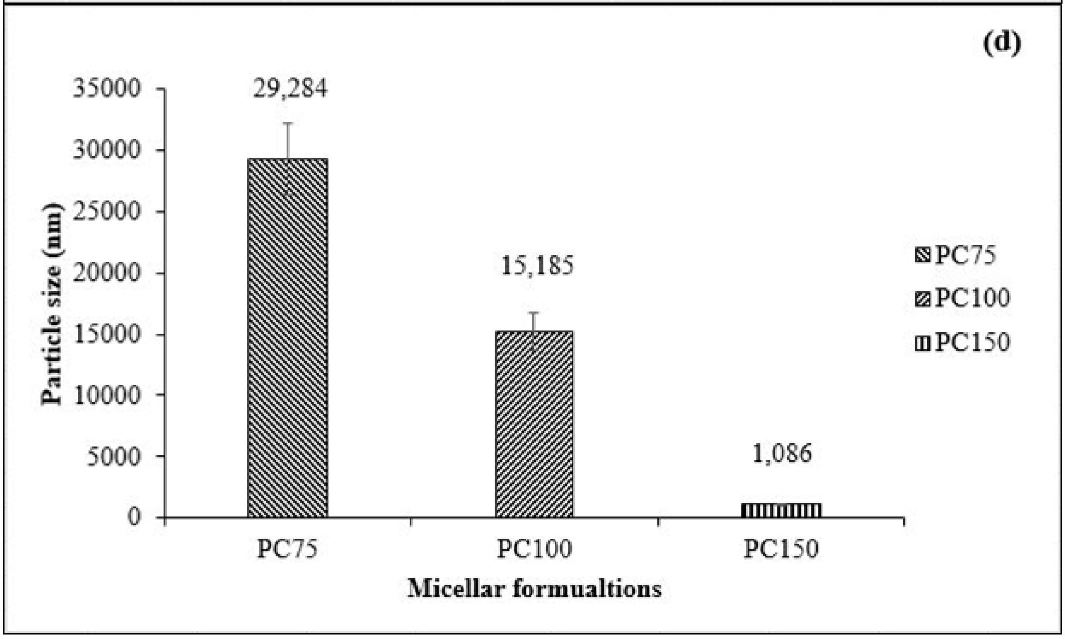
(d)
Particle size (nm)
35000
30000
25000
20000
15000
10000
5000
0
29,284
15,185
1,086
PC75
PC100
PC150
Micellar formualtions
PC75
PC100
PC150

Figure S7. The properties of the micelles: (**a**) the suspension solution of LA-loaded micelles; (**b**) the particle morphology of LA-loaded micelle ($PC_{75}LA$) observed under the upright microscopy, the scale bar is 10 μm; (**c**) the particle size of blank micelle and (**d**) the particle size of LA-loaded micelle determined by DLS (PDI < 0.3). Please refer to the section 3.2.2 for the results and discussion for this figure.

© 2016 by the authors. Submitted for possible open access publication under the terms and conditions of the Creative Commons Attribution (CC-BY) license (http://creativecommons.org/licenses/by/4.0/).
